# Annexin A2 Modulates ROS and Impacts Inflammatory Response via IL-17 Signaling in Polymicrobial Sepsis Mice

**DOI:** 10.1371/journal.ppat.1005743

**Published:** 2016-07-07

**Authors:** Sisi He, Xuefeng Li, Rongpeng Li, Lizhu Fang, Lingyun Sun, Yongsheng Wang, Min Wu

**Affiliations:** 1 State Key Laboratory of Biotherapy and Cancer Center, West China Hospital, Sichuan University, and Collaborative Innovation Center for Biotherapy, Chengdu, Sichuan, P. R. China; 2 Department of Biomedical Sciences, University of North Dakota, Grand Forks, North Dakota, United States of America; 3 Department of Rheumatology and Immunology, The Affiliated Drum Tower Hospital, Nanjing University Medical School, Nanjing, Jiangsu, P. R. China; Purdue University, UNITED STATES

## Abstract

Sepsis is a progressive disease manifesting excessive inflammatory responses, severe tissue injury, organ dysfunction, and, ultimately, mortality. Since currently, there are limited therapeutic options for this disease, further understanding the molecular pathogenesis of sepsis may help develop effective treatments. Here we identify a novel role for Annexin A2 (AnxA2), a multi-compartmental protein, in inhibiting pro-inflammatory response by regulating reactive oxygen species (ROS) and IL-17 signaling during sepsis. In cecal ligation and puncture (CLP) sepsis models, *anxa2* ^*-/-*^ mice manifested increased pro-inflammatory cytokines and neutrophil infiltration, but decreased bacterial clearance and animal survival. In addition, AnxA2 deficiency led to intensified ROS and IL-17A. Using site directed mutagenesis, we uncovered that cysteine 9 of AnxA2 was the most important aa (site) for regulation of ROS levels. Furthermore, ROS appears to be responsible for elevated IL-17A levels and subsequently exaggerated inflammatory response. Depletion of IL-17 via CRISPR/Cas9 KO strategy down-regulated inflammation and conferred protection against sepsis in *anxa2* ^*-/-*^ mice. Our findings reveal a previously undemonstrated function for AnxA2 in inflammatory response in polymicrobial sepsis models via an AnxA2-ROS-IL-17 axis, providing insight into the regulation of pathophysiology of sepsis.

## Author Summary

We determined the role of AnxA2 in sepsis based on cecal ligation and puncture (CLP). We demonstrated that AnxA2-deficient mice develop faster bacterial growth, more severe tissue injury and greater mortality compared to wild-type animals. The impaired bacterial control and higher mortality in the absence of AnxA2 are associated with greater neutrophil infiltration, ROS generation and IL-17A production, which are associated with oxidative sensing by cysteine 9 of AnxA2. Intensified IL-17A decreases bacterial clearance in this model although one of its primary roles is to assist in the clearance of translocating bacteria in the gut, in part by recruiting neutrophils to the disease sites. That said, in sepsis (at least in this animal model), there may be "too much of a good thing" and the negative consequences of this overzealous inflammatory response may become maladaptive. Fortunately, we demonstrate that AnxA2 as a novel regulator effectively contains the heightened inflammation through sensing and regulation of oxidation.

## Introduction

Severe sepsis is frequently associated with dysfunction and failure of critical organs, such as acute respiratory distress syndrome (ARDS), septic shock, and multiple organ dysfunction syndrome (MODS) [1]. Approximately 18 million new cases of severe sepsis occur each year globally and the incidence rate is still increasing at an alarming rate (8%) [2]. Unlike some of other epidemic illnesses, the current clinical treatment for sepsis is nonspecific and largely supportive, including support of organ function, and administration of fluids, antibiotics and oxygen [3]. To improve the clinical outcomes, further understanding the molecular details of sepsis is warranted. To better characterize pathophysiological and immunological features of sepsis, various animal models have been developed. Cecal ligation and puncture (CLP) is the most commonly-used model because it highly resembles human sepsis with high reproducibility [4].

Impaired responsiveness to pathogenic microbes and their products was frequently observed during sepsis [5]. Macrophages responding to infection were found to be reprogrammed [6]. T and B cells are also resistant to activation and proliferation signals under sepsis [7]. The number of circulating CD4^+^ T lymphocytes is increased in peripheral blood of septic patients, and the number of B and T cells is also decreased in the spleen [8]. The amount of CD4^+^CD25^+^ T-reg cells is increased during a sepsis process, whereas the expression of cytotoxic T lymphocyte associated antigen (CTLA)-4-D152, an inhibitory ligand, appears to be increased [9]. Additionally, myeloid-derived suppressor cells are also increased in sepsis. In the spleen and lung of serious sepsis patients, levels of programmed cell death 1 (PD-1) are increased [10]. Due to the complexity of disease processes, search for effective therapeutic strategies for sepsis is a daunting challenge, like finding a needle in a haystack.

A number of studies have demonstrated that many promising agents are effective on controlling animal sepsis, including low doses of corticosteroids, LPS-target agents and blockers of inflammatory molecules, as well as anti-HMGB1 (high mobility group box 1) antibody and anti-IL-17A antibody [11–14]. However, blocking proinflammatory cytokines in CLP-induced sepsis, such as IL-12 blockers and anti-TNF antibody, has been proven to have little or no benefit to patients [15,16]. Alternatively, some novel therapeutic approaches have been explored. For example, hydrogen gas is shown to increase survival rates in animals as oxidative stress and uncontrolled inflammatory response are pivotal to the progression of sepsis and may be promising targets [17]. Moreover, a number of candidate genes have been investigated in sepsis susceptibility, including protein C, macrophage inhibitory factor (MIF) and certain miRNAs [18]. Because of the heterogenic or largely ineffective outcomes from various therapeutic modalities, an improved understanding of sepsis pathophysiology, such as molecular pathogenesis by oxidative stress and inflammatory response, is urgently needed for developing better therapeutic strategies.

Annexins are known as a conserved family of Ca^2+^-regulated phospholipid-binding proteins and have existed over 500 million years [19]. Among its family members, AnxA2 is the most extensively studied [20,21]. AnxA2 is expressed in a variety of cells, such as tumor cells, endothelial cells, macrophages, and mononuclear cells. It is a multi-compartmental protein involved in a growing list of cellular processes and human diseases. In inflammatory dendritic cells, AnxA2 preserves late endosomal/lysosomal membrane integrity, thus modulating inflammation in arthritis [22]. Increased expression of AnxA2 has been observed in glioblastoma [23]. Hence, there appears to have multifaceted roles of AnxA2 in human health and disease. However, the relationship between AnxA2 and sepsis has not been clearly documented. Here, we used a sepsis model in *anxa2* ^*-/-*^ mice to evaluate whether AnxA2 exerted regulatory and organ protective functions based on CLP procedures. Our overall data suggest that AnxA2 indeed plays a critical role in inhibiting heightened inflammatory response by regulation of ROS and IL-17A in this experimental sepsis.

## Results

### AnxA2 deficiency aggravates host response to polymicrobial sepsis

**Fig 1:**
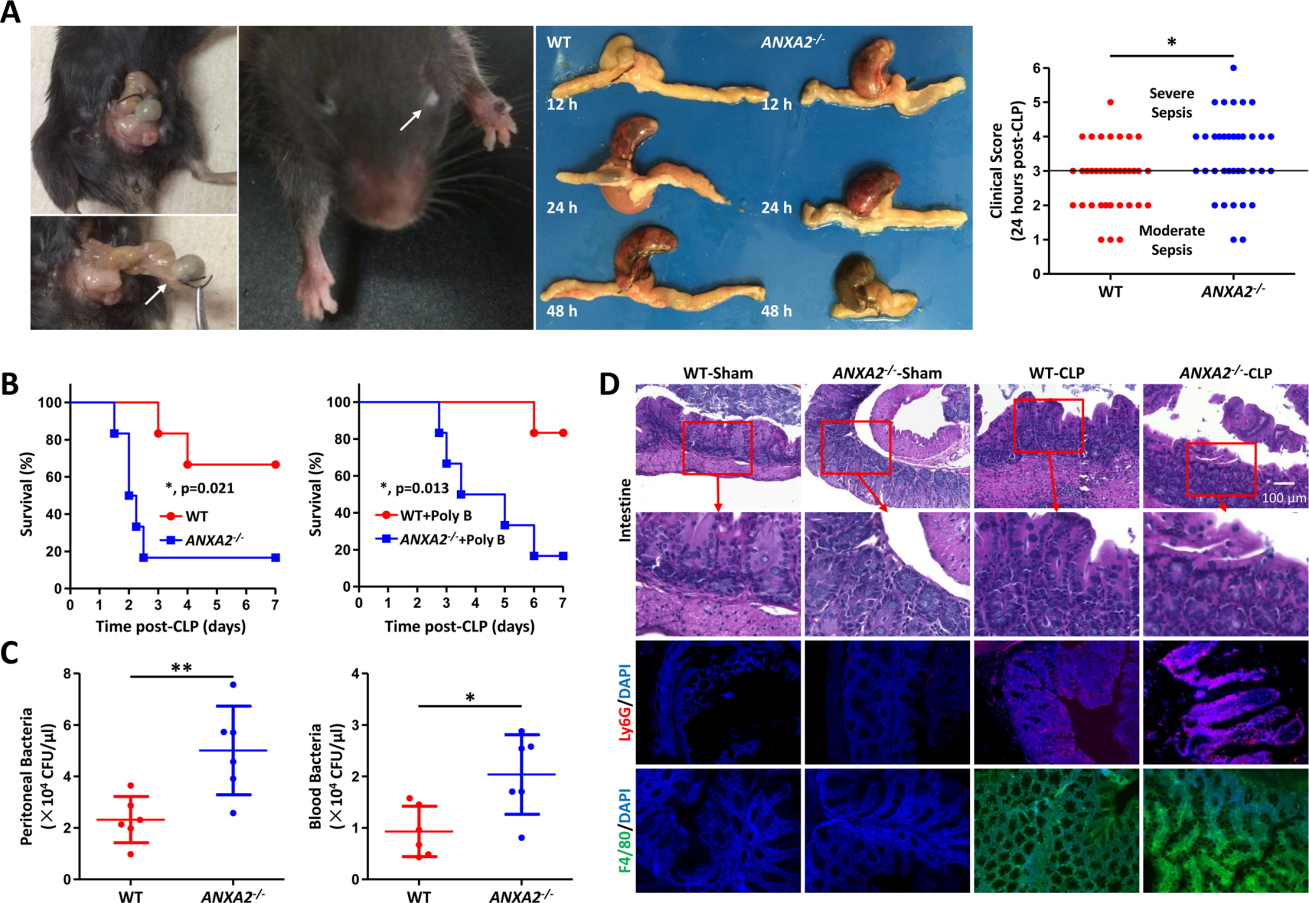
AnxA2 deficiency aggravates inflammatory response to polymicrobial sepsis. WT and *anxa2* ^*-/-*^ mice were subjected to CLP at time 0. (A) At 24 h post-CLP, mice were scored for the presence or absence of 6 different macroscopic signs of sepsis. A clinical score>3 is considered as severe sepsis. Data are shown as means±SD from 6 mice in each group. Left panels are representative macroscopic pictures from WT mice suffering sepsis. Middle panels are representative pictures of cecal ligation for different time points. (B) WT and *anxa2* ^*-/-*^ mice were also pretreated with polymyxin B (4 mg/kg) and then subjected to CLP 1 h later. Kaplan-Meier survival curves (n = 6, p<0.05, Log-rank Test). (C) 24 h after CLP, peritoneal lavage and blood were collected and plated for colony forming units (CFU). Means±SD, n = 6. (D) 24 h after CLP, colon tissues were paraffin-embedded for histological analysis. Ly6G and F4/80 were used to detect neutrophil and macrophage accumulation. Data are representative of three independent experiments. Scale bar = 5 μm. One-way ANOVA (Tukey’s post hoc); *, p<0.05; **, p<0.01.

Previously, we have found that AnxA2 is involved in Gram-negative bacterial infection [24,25]. To further understand whether AnxA2 has general immunity against a variety of pathogenic conditions, we employed the classical sepsis model in mice [26]. Induction of polymicrobial sepsis resulted in a worse phenotype in *anxa2* ^*-/-*^ mice. At 24 h post-CLP, WT mice developed moderate sepsis (78% of mice with clinical score≤3), whereas *anxa2* ^*-/-*^ mice exhibited worse sepsis (50% of mice scored>3) (Fig 1A). Following pretreatment with polymyxin B, *anxa2* ^*-/-*^ mice exhibited higher mortality rates vs. mock controls (Fig 1B). As sepsis is frequently preceded by bacteremia, we investigated whether *anxa2* ^*-/-*^ mice displayed altered bacterial clearance *in vivo*. As expected, *anxa2* ^*-/-*^ mice manifested higher peritoneal bacterial loads following CLP as quantified by colony forming units (CFU) (Fig 1C). Blood bacterial counts were also elevated in *anxa2* ^*-/-*^ mice (Fig 1C). We evaluated the extent of tissue structural damage and inflammatory response in *anxa2* ^*-/-*^ mice 24 h post-CLP and found that the integrity of colon tissues was severely destructed during CLP-induced sepsis in *anxa2* ^*-/-*^ mice (Fig 1D), which was accompanied with increased neutrophil infiltration and macrophage accumulation into damaged local areas (Fig 1D). All these phenomena imply an important role of AnxA2 in host defense in CLP-induced sepsis.

### Inflammatory cells and soluble mediators are increased in *anxa2* ^*-/-*^ mice upon CLP

**Fig 2:**
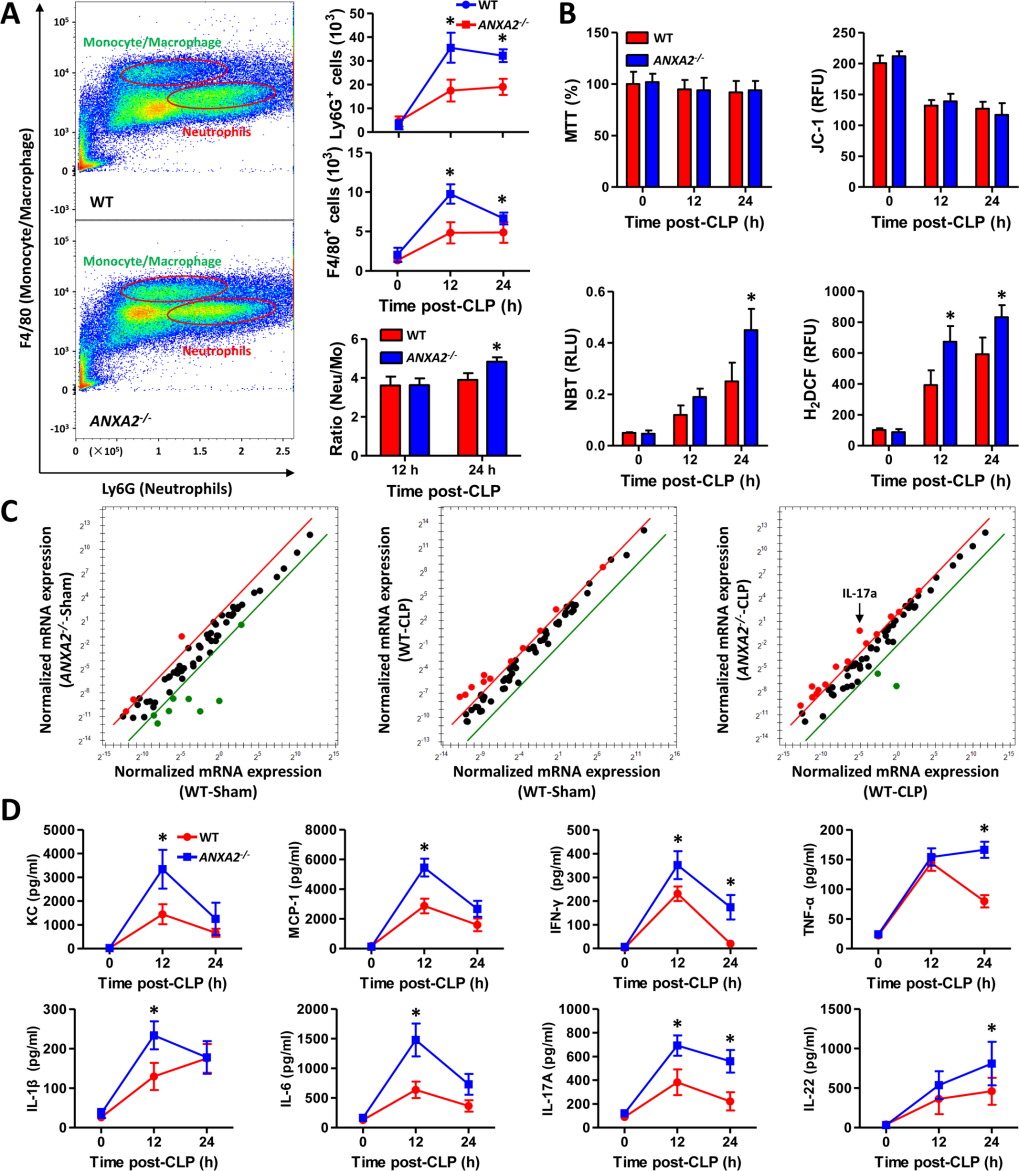
AnxA2 deficiency results in increased inflammatory response. (A) Scattergrams illustrating neutrophils (identified by Ly6G^+^F4/80^-^) and monocytes/macrophages (identified by Ly6G^-^F4/80^+^) positive events in peritoneal lavages from WT and *anxa2* ^*-/-*^ mice at 24 h post-CLP. Ly6G^+^ and F4/80^+^ cell, and ratio of neutrophils to monocytes were quantified and shown as means±SD. (B) Peritoneal macrophages (PMs) were collected to determine viabilities (MTT), mitochondrial potential (JC-1), intracellular O_2_(-) production (NBT), and generation of ROS (H_2_DCF), respectively. Means±SD from triplicate. (C) PMs were also subjected to inflammatory response based gene detection using real-time qPCR array. (D) ELISA detecting cytokine secretion in peritoneal lavage from mice at 12 h and 24 h post-CLP, means±SD from triplicate. Data are representative of three experiments, one-way ANOVA (Tukey’s post hoc); *, p<0.05.

To gain insight into the exaggerated inflammatory response, we next evaluated relevant cells for CLP. Analysis of peritoneal exudates by flow cytometry demonstrated increased peritoneal leukocyte accumulation at 12 h and 24 h post-CLP (S1A Fig). 24 h post-CLP, accumulation of Ly6G^+^ and F4/80^+^ cells was increased in *anxa2* ^*-/-*^ mice, and yielding a marked increase in the ratio of neutrophils to macrophages (Fig 2A). Analysis of T-lymphocyte and B-lymphocyte numbers revealed no alterations with deficiency of AnxA2 (S1B Fig). Macrophages from peritoneal lavage (PMs) were pelleted for cell viability test, and no differences were found in PMs of *anxa2* ^*-/-*^ mice post-CLP vs. WT mice (Fig 2B). The membrane-permeant JC-1 assay was also performed and no difference in PMs’ mitochondrial potential was observed between *anxa2* ^*-/-*^ mice and WT mice (Fig 2B). Nitroblue tetrazolium (NBT) and H_2_DCF assay measured reactive oxygen species (ROS), which was increased in PMs from *anxa2* ^*-/-*^ mice (Fig 2B). We then profiled inflammatory response genes in PM’s RNA by array-based analysis. A series of genes were found to be increased at 24 h post-CLP, with IL-17A being the most up-regulated in *anxa2* ^*-/-*^ mice (Fig 2C, S1 Table). The peritoneal lavage fluids were profiled for cytokine secretion and several pro-inflammatory cytokines (KC, IFN-γ, and TNF-α) were determined to be increased in *anxa2* ^*-/-*^ mice post-CLP (Fig 2D). In addition, cytokine levels (IL-6 and IL-17A) at the late stages (48 or 72 h) also increased in *anxa2* ^*-/-*^ mice as compared to WT mice, albeit less so than the early stages (S1C Fig).

### Distant organ injuries in CLP-induced polymicrobial sepsis

**Fig 3:**
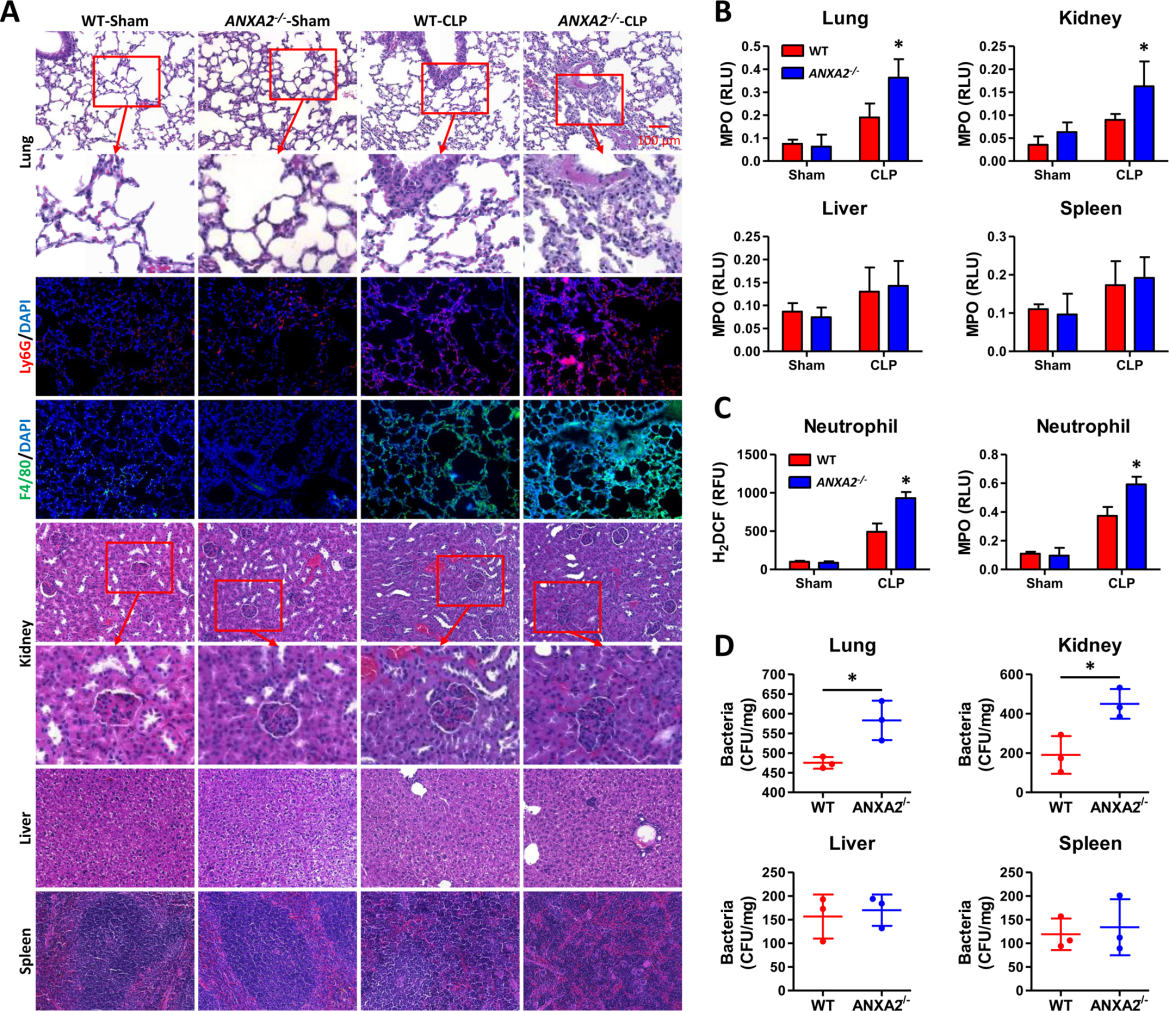
Distant organ injuries in mice with polymicrobial sepsis. (A) Mice were subjected to CLP procedures to induce polymicrobial sepsis. At 24 h post-CLP, lung, kidney, liver and spleen tissues were sectioned for H&E staining and the lung was immunostained with Ly6G or F4/80. (B) MPO determined in homogenates from above-indicated tissues. (C) 24 h after CLP, neutrophils were isolated from blood for H_2_DCF and MPO assays after 1 h incubation. (D) CFUs evaluated with the lung and kidney as above (n = 3, means±SD). A, data are representative of three independent experiments. B, C, means+SD from triplicate. One-way ANOVA (Tukey’s post hoc); *, p<0.05. Scale bar = 5 μm.

To explore whether AnxA2 deficiency contributes to distant organ injuries during the pathogenesis of the CLP model, we paraffin-embedded and sectioned lung, kidney, liver and spleens for histological analysis. During polymicrobial sepsis, *anxa2* ^*-/-*^ mice exhibited severe lung and kidney tissue damage without significant injury in the liver and spleen tissues (Fig 3A). We used Ly6G and F4/80 immunostaining to identify increased neutrophil infiltration and macrophage accumulation in *anxa2* ^*-/-*^ mice at 24 h post-CLP (Fig 3A, S2A Fig). The lung and kidney tissues of *anxa2* ^*-/-*^ mice also exhibited increased myeloperoxidase (MPO) levels, but the liver and spleen tissues did not (Fig 3B). H_2_DCF and MPO assays determined oxidative intensity (ROS) of neutrophils and PMs, which showed increased oxidation in *anxa2* ^*-/-*^ mice upon CLP treatment (Fig 3C, S2B Fig). We assessed a serological marker of kidney and liver damage by alanine aminotransferase (ALT) assay and found increased ALT in *anxa2* ^*-/-*^ mice upon CLP treatment (S2C Fig). Furthermore, bacterial loads in the lung and kidney of *anxa2* ^*-/-*^ mice have increased, but CFU in the liver and spleen did not (Fig 3D). These data collectively indicate that, besides the colon, the lung and kidney are the main target organs with sepsis injury.

### AnxA2 deficiency increased ROS and inflammatory responses upon CLP

**Fig 4:**
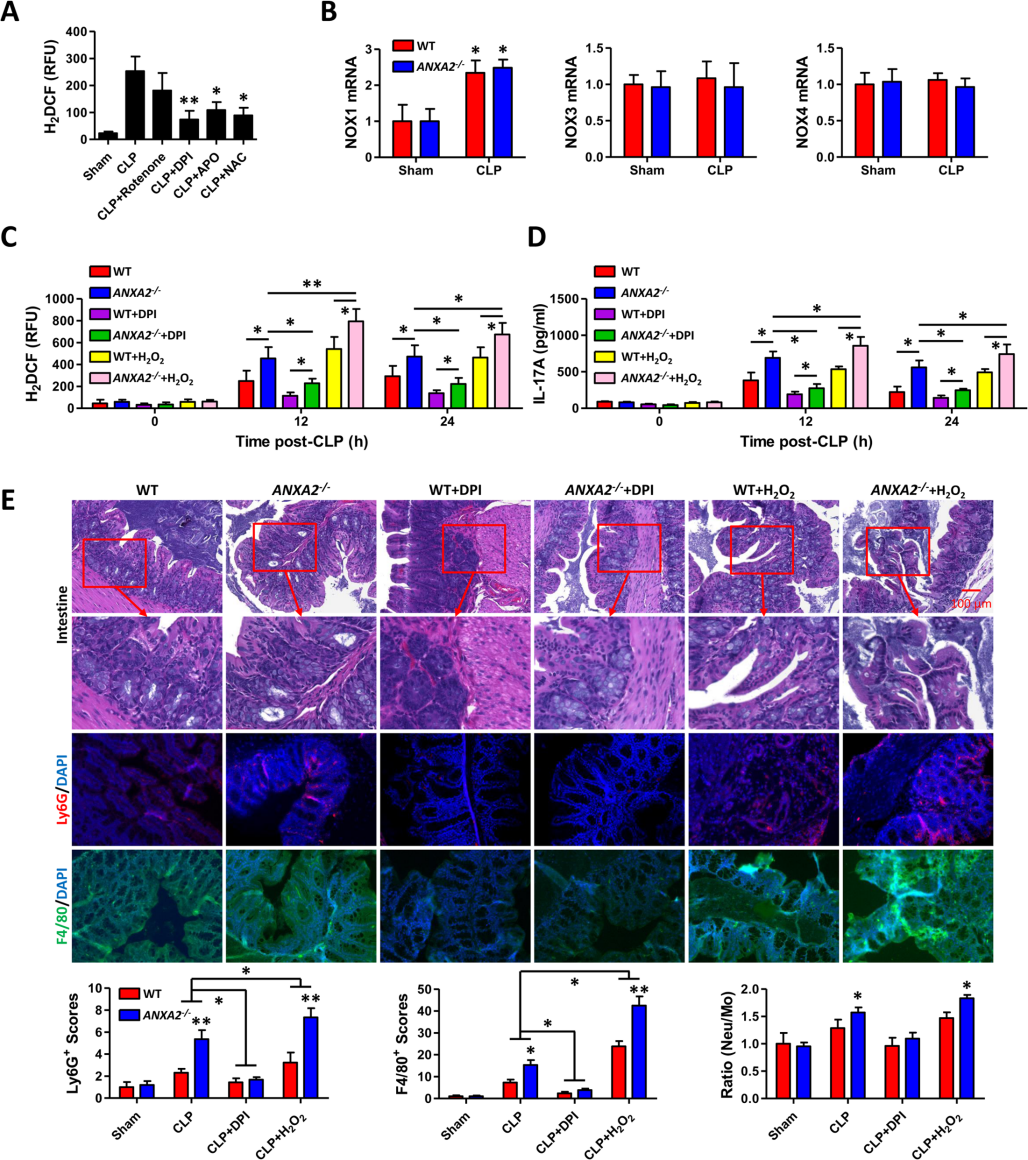
Increased ROS levels and inflammatory responses in *anxa2* ^*-/-*^ mice with polymicrobial sepsis. (A) ROS levels were detected in PMs from *anxa2* ^*-/-*^ mice pre-injected with rotenone, DPI, APO, and NAC, respectively. (B) PMs from WT and *anxa2* ^*-/-*^ mice were subjected to qPCR to detect the NOX1, NOX3, and NOX4 expression. (C) Mice were pretreated with H_2_O_2_ or DPI and at 12 h or 24 h post-CLP, ROS levels in PMs were detected. (D) IL-17A in peritoneal lavage was assayed by ELISA. (E) At 24 h post-CLP, colon tissues were stained for histological analysis. Ly6G and IL-17A were used to detect immune cells recruitment and inflammatory cytokine secretion. Data are representative of three experiments and expressed as means+SD from triplicate. One-way ANOVA (Tukey’s post hoc); *, p<0.05; **, p<0.01. Scale bar = 5 μm.

In addition to their toxic properties, ROS are key signaling mediators and play important roles in the progression of inflammatory disorders [27]. To elucidate whether AnxA2 plays a role in ROS-mediated inflammatory response upon CLP, we searched for the origin of CLP-induced ROS production. With pretreatment of several ROS inhibitors indicated below, PMs from WT mice showed decreased ROS levels at 24 h post-CLP. The lowest ROS levels were found in the diphenyleneiodonium (DPI)-treated group; however, rotenone, apocynin (APO), and N-acetylcysteine (NAC) hardly decreased ROS levels (Fig 4A). This suggests that the source of ROS may be NADPH oxidase instead of mitochondria [28]. In addition, we found that DPI reduced peritoneal bacteria upon CLP treatment (S3A Fig). Further, we transfected p47^*phox*^ S303A/S304A (serine mutated to alanine) plasmid into WT or *anxa2* ^*-/-*^ mice, and found that ROS production in neutrophils and PMs was only increased in control vector-transfected mice after CLP procedure (S3B and S3C Fig). We next evaluated the quantity of various NOX isoforms in PMs using quantitative real-time PCR. While 24 h post-CLP did not significantly change NOX3 and NOX4 levels, NOX1 was markedly increased with a magnitude over 2 folds (Fig 4B), meaning that NOX1 may be the primary source of ROS. DPI and H_2_O_2_ pretreatment down-regulated and up-regulated ROS levels, respectively, in both WT mice and *anxa2* ^*-/-*^ mice. We also found higher ROS and IL-17A cytokine levels in *anxa2* ^*-/-*^ mice than WT mice at both 12 h and 24 h (Fig 4C and 4D). Further, ROS compromised bacterial burdens as determined by CFU (S4A Fig). DPI also contributed to better survival of mice during CLP-induced sepsis, while H_2_O_2_ hampered mouse survival (S4B Fig). Next, colon tissues were subjected to histological analysis to dissect tissue damage upon CLP-induced sepsis. Severe tissue injury was found in *anxa2* ^*-/-*^ mice, and was further aggravated by H_2_O_2_ (Fig 4E). Macrophages and neutrophils were shown to be accumulated in the injured areas and positively correlated with ROS levels. Furthermore, DPI decreased while H_2_O_2_ increased the accumulation of Ly6G and F4/80 positive cells (Fig 4E). Finally, the ratio of neutrophils to monocytes is higher upon H_2_O_2_ treatment, indicating further intensified inflammatory response (Fig 4E).

### Neutrophils and monocytes are main sources of IL-17A upon CLP

**Fig 5:**
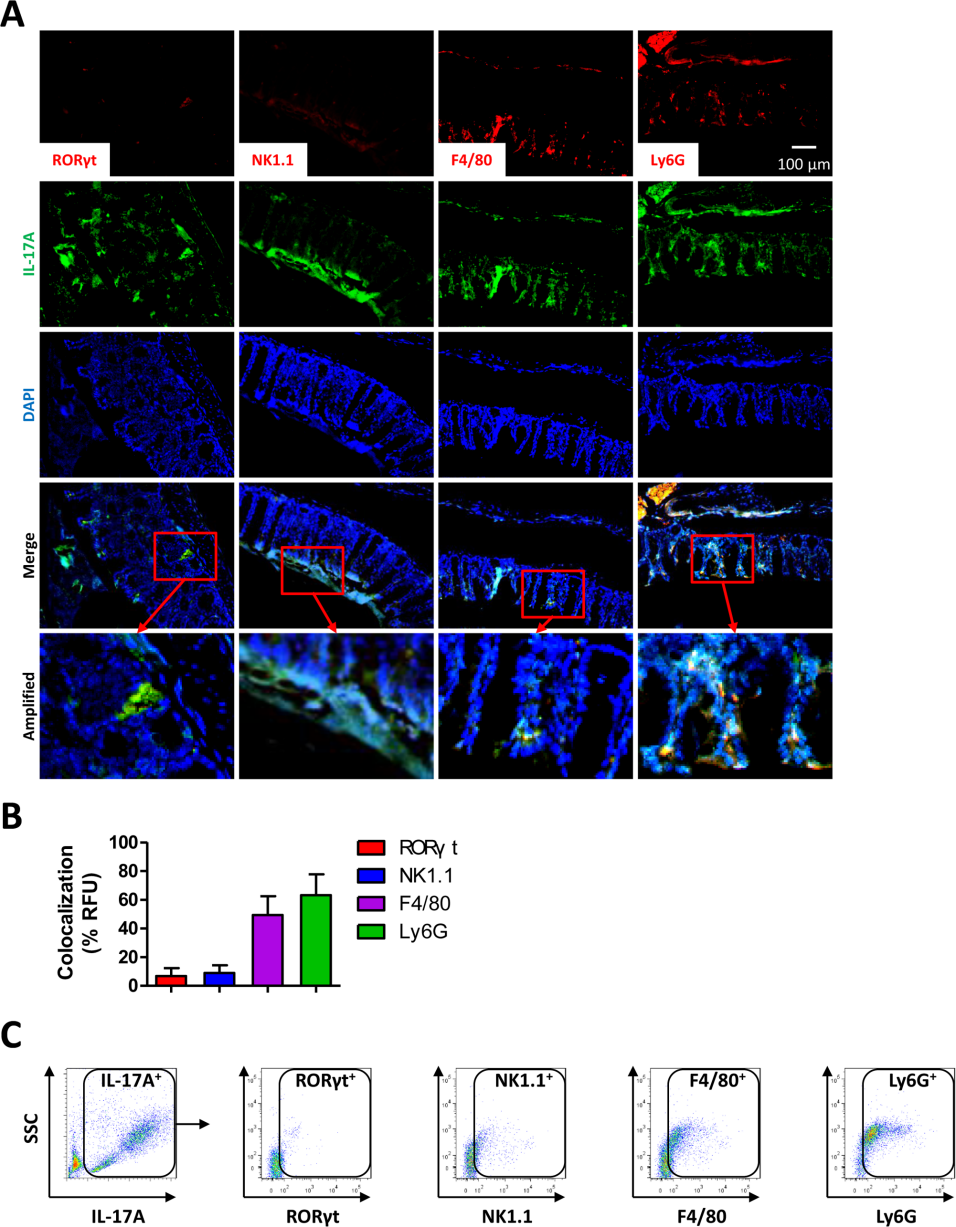
Neutrophils and monocytes are main sources of IL-17A upon CLP. (A) *anxa2* ^*-/-*^ mice were subjected to CLP procedures to induce polymicrobial sepsis. At 24 h post-CLP, colon tissues were sectioned for immunostaining with RORγt, NK1.1, F4/80, Ly6G, and IL-17A antibodies. (B) Colocalization of red fluorescence (to label indicated cell types) with green fluorescence (IL-17A^+^) quantified as percentage of total green fluorescence. (C) IL-17A positive events in peritoneal lavages from *anxa2* ^*-/-*^ mice at 24 h post-CLP. The IL-17A^+^ cells were collected and further stained with different antibodies and shown as scattergrams. Data are representative from 3 independent experiments. Scale bar = 100 μm.

Since IL-17A is the most up-regulated in PMs of *anxa2* ^*-/-*^ mice at 24 h post-CLP, we next investigated cell sources for IL-17A production by analyzing ILCs, NK/NKT cells, monocytes, and neutrophils in CLP tissues. Immunostaining determined the colocalization of IL-17A and related cell markers in colon tissues from *anxa2* ^*-/-*^ mice at 24 h post CLP procedures. As shown in Fig 5A and 5B, monocytes and neutrophils exhibited higher IL-17A than T cells or NK/NKT cells in colon tissues. This is consistent with flow cytometry data with heightened neutrophils and monocytes (Fig 5C), suggesting that IL-17A may be mainly derived from neutrophils and possibly also monocytes. Next, we used DPI and H_2_O_2_ to analyze whether IL-17A signaling is related to ROS levels. Increased ROS levels by H_2_O_2_ resulted in enhanced IL-17A secretion at 24 h post-CLP (S5A Fig). In addition, *anxa2* ^*-/-*^ mice exhibited higher IL-17A secretion than WT mice upon CLP procedures (S5A and S5B Fig). IL-17A in serum or peritoneal lavage was similarly increased to ROS in mice transfected with vector controls but not p47^*phox*^ S303A/S304A (DN) plasmid groups (S5C Fig). Furthermore, peritoneal bacteria in *anxa2* ^*-/-*^ mice with CLP were decreased by DN plasmid transfection (S5D Fig). These data collectively imply that AnxA2 deficiency increases ROS, which may affect inflammatory responses in the CLP model.

### AnxA2 C9A (cysteine to alanine) mutation fails to eliminate ROS upon CLP

**Fig 6:**
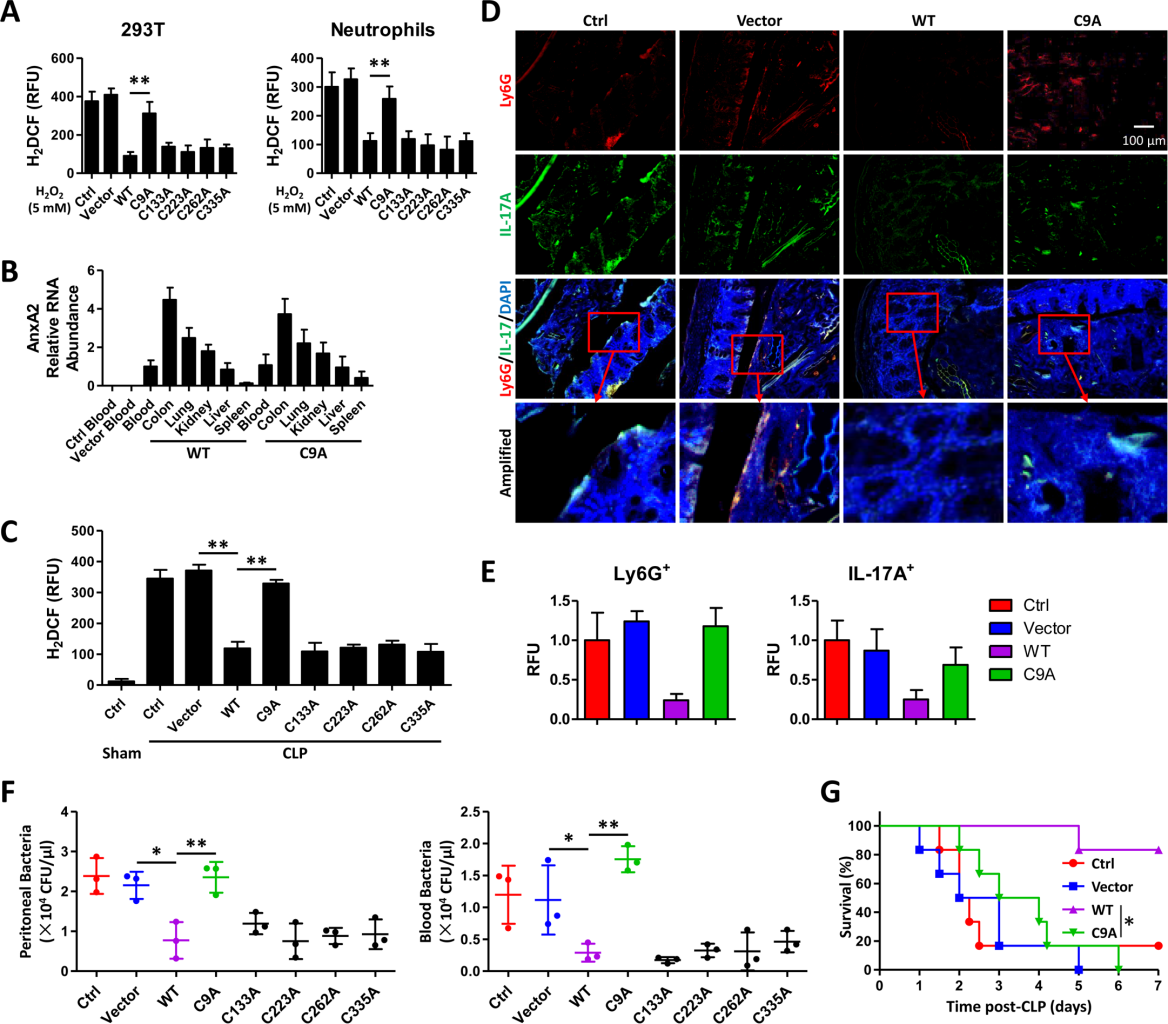
AnxA2 C9A mutation exacerbates host immunity in polymicrobial sepsis mice. (A) 293T cells and neutrophils from *anxa2* ^*-/-*^ mice were transfected with AnxA2 WT, C9A, C133A, C223A, C262A, and C335A plasmids, respectively. After H_2_O_2_ treatment (5 mM, 30 min), ROS levels were measured using H_2_DCF assay. (B) *anxa2* ^*-/-*^ mice were tail vein injected with these plasmids, respectively. 24 h later, mice were sacrificed and AnxA2 mRNA relative abundance in different organs or tissues were measured. (C) After plasmids transfection successfully, the mice were subjected to polymicrobial sepsis, and ROS levels were detected in PMs from mice at 24 h post-CLP. Means+SD from triplicate. (D, E) Immunostaining was performed for Ly6G^+^IL-17A^+^ cell accumulation detection in colon tissues from mice above. Fluorescence scores were quantified. (F) Peritoneal and blood bacteria were determined using CFU assay, means±SD from 3 mice. (G) Survival of mice (n = 6) above with polymicrobial sepsis was monitored. Kaplan-Meier survival curves (p = 0.0016, Log-rank Test). A-C, means+SD from 3 mice. D, E, data are representative from three independent experiments. One-way ANOVA (Tukey’s post hoc); *, p<0.05; **, p<0.01. Scale bar = 5 μm.

A previous study suggests that elevated NOX1 mRNA levels likely contribute to higher ROS production [29]. However, CLP procedures did not induce obvious difference in NOX1 levels between WT mice and *anxa2* ^*-/-*^ mice (Fig 4B). Thus, we attempted to determine whether ROS are regulated at post-transcriptional levels by AnxA2. Annexins are a structurally related family of calcium and phospholipid-binding proteins, which regulate a wide range of cellular activities [22]. AnxA2 possesses redox sensitive cysteine(s), thus depletion of AnxA2 results in elevated ROS upon oxidative stress, increased activation of ROS-induced pro-apoptotic kinases (JNK, p38 and Akt), and elevated sensitivity to ROS-mediated cellular damage/death [30]. Previous literature suggests that cysteines are critical in sensing and regulating oxidation [31,32]. To this end, we have generated AnxA2 WT overexpression plasmid and five AnxA2 site specific mutation plasmids (C9A, C133A, C223A, C262A, and C335A, cysteine mutated to alanine). 293T cells and neutrophils from *anxa2* ^*-/-*^ mice were transfected with these plasmids, respectively. After H_2_O_2_ treatment, ROS levels were measured using H_2_DCF assay. We found that transfection of WT, C133A, C223A, C262A, and C335A plasmids could reduce ROS in these two cells, while negative ctrl, empty vector control and C9A groups could not (Fig 6A). These results mean that this cysteine (C9) is indeed oxidized in AnxA2 protein. These plasmids were complexed with jetPEI and were then tail vein injected to mice 24 h prior to CLP procedures, and AnxA2 protein abundance in colon tissue was measured using immunoblotting, which showed that plasmid-encoded AnxA2 variants are indeed expressed *in vivo* both in WT and *anxa2* ^*-/-*^ mice in our experiments (S6A Fig). AnxA2 mRNA could be found in blood, colon, lung, kidney, liver, and spleen 24 h after CLP, while WT or C9A plasmid introduction did not affect the expression in these organs (Fig 6B). Interestingly, we found more AnxA2 mRNA abundance in colon tissue and less so in spleens or other organs (Fig 6B). Further, PMs from *anxa2* ^*-/-*^ mice were isolated to determine ROS 24 h post-CLP. Mice treated with C9A plasmid showed higher ROS levels than mice injected with WT or other AnxA2 mutation plasmids (C133A, C223A, C262A, and C335A) that target to various regions in this protein (Fig 6C), suggesting that cysteine 9 of AnxA2 is the most important site for oxidation sensing. IL-17A and other cytokines (TNF-α, IL-6, IL-22) in peritoneal lavage from above-treated mice were analyzed by ELISA. Correlated with ROS levels, C9A plasmid pre-injection led to increased cytokines compared to WT plasmid or other mutation plasmid (S6B Fig). Importantly, AnxA2 WT plasmid injection decreased the accumulation of Ly6G^+^ cells, while C9A plasmid failed to do so (Fig 6D and 6E). Mice received AnxA2 WT plasmid and other four mutation plasmids showed lower peritoneal and blood bacterial burdens than those received AnxA2 C9A plasmid at 24 h post-CLP (Fig 6F). Importantly, mouse survival was monitored for seven days post-CLP and AnxA2 WT plasmid injection effectively rescued mice from mortality (Fig 6G).

To investigate whether AnxA2 generally affects ROS levels in other sepsis models, we employed bacterial infection models with *E*. *coli* and *P*. *aeruginosa* to study the relationship between ROS levels and IL-17A release. At 12 h and 24 h post-infection, both *E*. *coli* and *P*. *aeruginosa* resulted in elevated ROS levels and IL-17A production under AnxA2 deficiency (S7A and S7B Fig). C9A mutation failed to decrease IL-17A production upon *E*. *coli*-induced sepsis determined by immunostaining (S7C and S7D Fig). Similarly, AnxA2 C9A plasmid failed to decrease ROS levels in *anxa2* ^*-/-*^ mice with bacterial sepsis (S7E Fig), and injection of AnxA2 WT plasmid partially reduced IL-17A levels in *anxa2* ^*-/-*^ mice after infection (S6F Fig). These data together imply that the important role of AnxA2 in inflammatory response is also dependent on cysteine 9 residue to control ROS levels in other models including bacterial infection.

### AnxA2 impedes ROS-mediated IL-17 signaling in CLP mice

**Fig 7:**
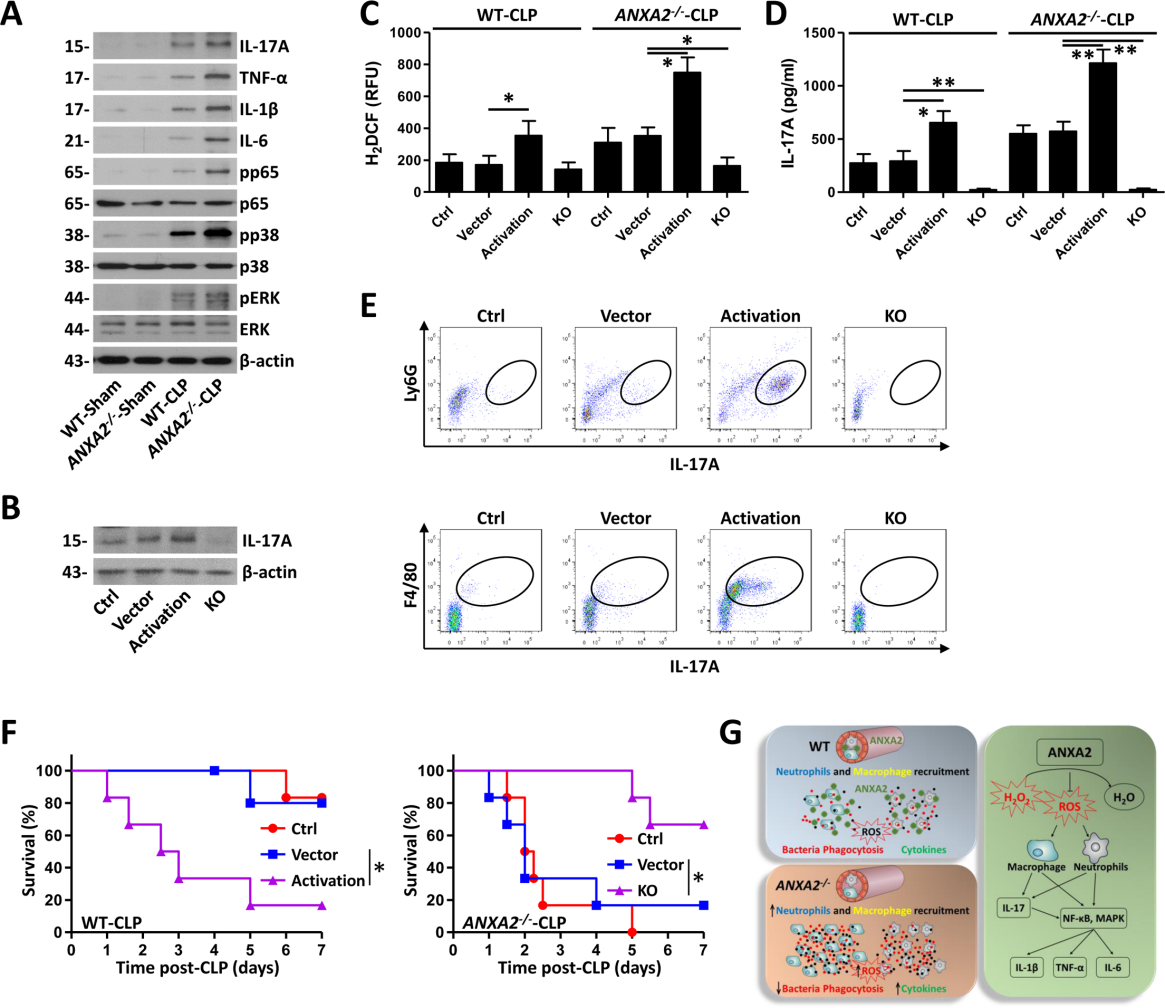
Elevated IL-17 signaling in *anxa2* ^*-/-*^ mice with polymicrobial sepsis. (A) Mice were subjected to CLP for 24 h. PMs isolated from peritoneal lavage were homogenized for immunoblotting of IL-17 signaling proteins. Data are representative from 3 independent experiments. (B) WT mice were pre-tail vein injected with IL-17 activation and KO plasmids, and 24 h later subjected to CLP-induced sepsis for 24 h. PMs isolated from peritoneal lavage were homogenized for immunoblotting of IL-17A. Data are representative from 3 independent experiments. (C) Mice were pre-tail vein injected with the IL-17 activation or KO plasmid, and 24 h later subjected to CLP-induced sepsis for another 24 h. ROS levels in PMs were determined using H_2_DCF assay. (D) IL-17A in peritoneal lavage fluid was assayed by ELISA. Means+SD from triplicate. (E) F4/80^+^IL-17A^+^ and Ly6G^+^IL-17A^+^ cells from peritoneal lavage of WT mice were enumerated by flow cytometry. (F) Survival of mice in CLP sepsis in the presence of the IL-17 activation or KO plasmid. Kaplan-Meier survival curves (n = 6, p<0.05, Log-rank Test). (G) Loss of host defense against polymicrobial sepsis in the absence of AnxA2. Data are representative of three independent experiments. One-way ANOVA (Tukey’s post hoc); *, p<0.05. Scale bar = 5 μm.

To further dissect the role of IL-17A in AnxA2-mediated signals in the CLP model, we evaluated proinflammatory effects due to IL-17 signaling. Immunoblotting showed that levels of TNF-α, IL-1β, and IL-6 were increased in PMs at 24 h post-CLP, which were further increased in *anxa2* ^*-/-*^ mice (Fig 7A, S8A Fig). In addition, several IL-17A downstream signals, such as NF-κB and MAPK, were found to be activated upon CLP, which were also further enhanced during AnxA2 deficiency (Fig 7A, S8A Fig). Because IL-17A plays an important role in neutrophil- or macrophage-infiltration, we investigated the role of IL-17A in polymicrobial sepsis. WT mice were injected with the IL-17 activation or KO plasmid, IL-17A expression was measured using immunoblotting and qRT-PCR (Fig 7B, S8B Fig). At 24 h post-CLP, IL-17A mRNA increased significantly in colon vs. other tissues (blood, lung, kidney, liver, and spleen), which may reflect more serious injury or inflammation in colon tissue (S8C Fig). In addition, manipulation of IL-17A partially altered ROS levels and IL-17A cytokine release in peritoneal lavage from mice upon CLP treatment (Fig 7C and 7D). While IL-17 plasmid transfection could not affect the recruitment of neutrophils and monocytes/macrophages in peritoneal lavage from normal WT mice (S8D Fig), Ly6G^+^IL-17A^+^ and F4/80^+^ IL-17A^+^ cells were increased by IL-17 activation and decreased by IL-17 KO, respectively (Fig 7E, S8E Fig). Bacterial burdens in peritoneal lavage and blood were also found to be decreased by IL-17 KO, indicating that treatment with the IL-17 KO plasmid promotes bacterial clearance (S8F Fig). Next, survival rates were monitored and the IL-17 activation plasmid was found to increase mortality upon CLP treatment, while the IL-17 KO plasmid decreased the mortality of sepsis mice (Fig 7F). These results indicate that IL-17A may play a role in inflammatory responses upon CLP procedures. Fig 7G proposes a novel model whereby AnxA2 regulates IL-17 signaling by targeting ROS. The local immune response by host cells appears to impact bacterial loads, whereas modulation of circulating mediators and distant organ functionality are primarily regulated by ROS production upon sepsis.

## Discussion

In this study, we identify AnxA2 as a critical mediator in host defense against sepsis using a mouse CLP model. *anxa2* ^*-/-*^ mice manifest a severe sepsis phenotype, including excessive macrophages and neutrophils, decreased survival, increased inflammatory response, impaired bacterial clearance, and apparent tissue injuries. A number of studies have shown that cysteine has a critical function in oxidative sensing in various settings [30,31,33]. Importantly, AnxA2 functions as a putative ROS inhibitor in sepsis progression, and cysteine 9 of AnxA2 is the most important aa for oxidation regulation, helping the host copes with the disease. Thus, AnxA2 may mitigate the severity of sepsis by modulating ROS and IL-17A levels. Our data also indicate that increased IL-17A is positively correlated with increased levels of ROS in *anxa2* ^*-/-*^ mice, and that further strategy with an IL-17 KO plasmid inhibits inflammatory response in sepsis mice.

As an intracellular protein that participates in cytoplasmic membrane-associated processes, AnxA2 is involved in diverse cellular processes including regulation of innate immunity, endosome function and inflammation [24]. Experimental evidence shows that AnxA2 is critical for down-regulation of inflammatory events [21]. There is report that Sirt1 activation markedly alters transcription profiles and improves outcome in experimental sepsis [34], which is similar to what we observed with AnxA2. Existing data imply that eliminating ROS is cytoprotective against sepsis [35]. Our data are consistent with previous studies that phagocyte-produced ROS may affect the disease progression, such as rheumatoid arthritis [36]. In addition, we also show that increased production of ROS was detected in whole peritoneal lavage of *anxa2* ^*-/-*^ mice in both CLP model and bacterial infection model (*E*. *coli* and *P*. *aeruginosa*). Similarly, recent work showed that AnxA2 down regulates oxidized cellular proteins or prevents ROS production in tissues, which may explain why ROS are increased in *anxa2* ^*-/-*^ mice [30]. Consistent with existing reports, we observed enhanced neutrophil infiltration, increased ratios of neutrophils to monocytes, and impaired bacterial clearance in sepsis mice [37–41]. Exacerbated activation of neutrophils can inflict tissue damage [42]. Targeting neutrophils in sepsis may have the potential for therapeutic intervention. Thus, AnxA2 deficiency-induced neutrophil infiltration may contribute to severe local and distant organ injury and higher mortality of sepsis mice.

Another important finding of this work is that IL-17 signaling is regulated by ROS, whereas inflammation was reduced after IL-17 knockdown. Deficiency of p47phox (a subunit of the NADPH oxidase) in mice is one of the animal models for chronic granulomatous disease (CGD), in which patients commonly inherits abnormalities of Nox2, p22phox, or p67phox and displays persistent inflammation in many tissues [43]. Recent studies indicated that restoration of Park7 expression rescues ROS production and improves survival in LPS-induced sepsis [35]. Through its C-terminus, Park7 binds to p47phox to promote NADPH oxidase-dependent production of ROS. In order to determine the origin of ROS in the CLP model, we used several of ROS inhibitors such as rotenone, DPI, APO, and NAC and found that ROS was markedly inhibited only in the DPI-treated group. This suggests that the source of ROS is probably from NADPH oxidase instead of mitochondria because DPI mainly inhibit ROS originated from NADPH [28]. Although DPI may have complex roles in mammalian cells, our data demonstrate that DPI plays a predominant role in inhibition of ROS derived from NADPH in our model. This is supported as blocking mitochondrial ROS did not impede oxidation levels [44]. However, we cannot exclude the possible involvement of Nrf2 pathways [45,46], which may be worth further studying. Furthermore, we transfected the p47^*phox*^ S303A/S304A plasmid into WT or *anxa2* ^*-/-*^ mice, and observed that ROS production in neutrophils and PMs was only increased in control vector-transfected mice after CLP procedures. This strongly supports that NADPH is critically involved in ROS production during the CLP process [35]. Together, our study shows that Anxa2 possesses a protective activity against sepsis by controlling NAPDH oxidase activation.

IL-17A is the signature cytokine in various diseases, particularly the development of autoimmunity, inflammation, and tumors, and also plays important roles in host defense against bacterial and fungal infections [47]. T_H_17 cells preferentially produce IL-17A, IL-17F, IL-21, and IL-22 [48]. We show that IL-17-mediated inflammation correlated with production of inflammatory cytokines (such as IL-6, IL-1β and TNF-α) that were profoundly increased at the early times upon infection including CLP treatment. At the late times, these cytokines petered off to relatively lower levels, which suits the host defense patterns and avoid unnecessary damage of critical organs such as the lung and liver. Thus IL-17A may contribute to sepsis progression, like IL-33 and CRTH2, which are potential therapeutic targets for polymicrobial sepsis [38,49]. Robust immunity against infection can only be achieved by a balanced inflammation. Previous studies showed that γδ T cells are major producers of IL-17A in the gut [50,51]. Others reported that acute-phase deaths from murine polymicrobial sepsis were characterized by innate immune suppression rather than exhaustion of the overall immunity [52]. In this sepsis model, other "innate" cells like ILCs and NK/NKT cells, monocytes and even neutrophils may produce IL-17A [53]. It was also reported that CLP markedly altered subsequent B-cell responses in the later process. Total IgG and IgM levels, as well as the memory B-cell response, were increased in septic mice [54]. Therefore we cannot ignore that the adaptive immunity including T_H_2 lineage may affect late processes in the CLP model [55]. Our data indicated that the monocytes and neutrophils seemingly produced more IL-17A in colon tissues than T cells or NK/NKT cells, which may be due to higher cell populations in the diseased regions and confirmed them as major IL-17A producing cells at the early phase in CLP.

We also uncovered that ROS levels are inversely correlated with AnxA2. To probe the molecular mechanisms by which AnxA2 controls ROS levels, we reasoned that cysteine in AnxA2 plays critical roles in oxidative sensing. To address this question, we created a series of mutants at various cysteine sites and evaluated the mutation that may impact oxidation sensing. Based on both *in vitro* and *in vivo* experimentation, we found that introduction of the AnxA2 C9A plasmid led to increased IL-17A, whereas introduction of the AnxA2 WT plasmid or other less critical cysteine mutation plasmids did not. These data strongly attest that cysteine 9 in AnxA2 may be a critical molecular base for this protein to generate strong oxidative sensing and efficiently regulate ROS production.

Our data are supported by other reports that IL-17A is induced by the NAD(P)H-oxidase dependent generation of ROS, leading to a pro-inflammatory activation in atherosclerosis [47]. The percentage of IL-17A^+^Ly6G^+^ cell population in *anxa2* ^*-/-*^ mice turned to be higher than WT mice, suggesting that neutrophils may play vital roles in mediating severe inflammation in *anxa2* ^*-/-*^ mice. We hypothesized that ROS can enhance secretion of IL-17A during sepsis and that hence immune cells may be critical mediators of host responses to sepsis [36,56]. However, these findings were counterintuitive with a clinical report that showed absolute counts of T_H_17 and T-reg cells in sepsis survivors were higher than non-survivors [39]. It is also interesting that the pattern of end organ injury is not uniform, with the liver and spleen being largely spared. As liver injury is a common feature of sepsis in humans. The difference may be due to species specific responses between mice and humans, suggesting that the CLP model may not quite exactly model the human sepsis [6,57].

In summary, we identify a non-redundant function of endogenous AnxA2 in host defense. AnxA2 exerts a protective role by multifaceted biological activities, especially regulation of ROS production and IL-17 signaling. This immunity function impacts both the original site and distal organs of the disease. Collectively, our findings indicate that AnxA2 may represent a new layer of host defense systems against bacterial infection, thus a potential therapeutic target for sepsis.

## Materials and Methods

### Ethics statement

This study was carried out in accordance with the recommendations of the Guide for the Care and Use of Laboratory Animals of the National Institutes of Health. The protocols were approved by the Institutional Animal Care and Use Committee (IACUC) at the University of North Dakota (Assurance Number: A3917-01). Animal procedures including operations and injections were performed under anesthesia using ketamine (40 mg/kg), and were in accordance with the ARRIVE reporting guidelines [58].

### Mouse model of cecal ligation and puncture (CLP)

Female C57BL/6J mice aged approximately 6–8 weeks were obtained from Jackson Laboratory (Bar Harbor, ME), and *anxa2* ^*-/-*^ mice on a C57BL/6J background were kindly provided by Dr. K. Hajjar (Cornell University, Ithaca, NY) [26]. Mice were maintained in the animal facility at University of North Dakota. A sublethal model of CLP was used according to a description published previously [59]. In brief, mice were anesthetized intraperitoneally with ketamine (40 mg/kg), and the abdominal area was shaved and disinfected. Then the cecum was identified and exposed, ligatured at its external third, and punctured with 27-gauge needle. Next the abdominal musculature and abdominal skin were closed by applying simple suture. In sham-surgery control mice, the cecum was only exposed but not punctured and was then returned to the abdominal cavity.

In order to monitor the health condition of experimental mice, we use a clinical score to evaluate the symptoms reflecting murine sepsis. The maximum score of 6 comprised the presence of the following signs: lethargy, piloerection, tremor, periorbital exudates, respiratory distress, and diarrhea. Mice with a clinical score >3 were defined as exhibiting severe sepsis; otherwise mice underlying score <3 were exhibiting moderate inflammatory response [37,38].

### Bacteria

*P*. *aeruginosa* wild-type (WT) strain PAO1 was a gift from Dr. Stephen Lory (Harvard Medical School, Boston, MA). *E*. *coli* (ATCC 25922) was bought from ATCC and NEB 5-alpha F'Iq Competent *E*. *coli* (C2992H) was bought from BioLabs Inc. Homogenates of peritoneal, blood, and other tissues were plated for colony forming units (CFU) assay [58,60].

### Plasmid construction

AnxA2 relevant genes were amplified from mice cDNA with specific primers by PCR and cloned into the PstI and XbaI sites of the PCAGGS-GFP vector (Addgene, Cambridge, MA) (S2 Table). Constructed plasmids were electroporated into DH5α strain using an Electroporator 2510 system (settings: 25 μF, 200 Ω, 2.5 kV; Eppendorf, Hauppauge, NY). Transformants were selected and maintained in LB medium containing 100 μM ampicillin (Sigma-Aldrich, St. Louis, MO). All of the nuclease, polymerase and ligase used in molecular cloning were bought from New England BioLabs Inc. Mice were tail-vein injected with vehicle Ctrl (*in vivo*-jetPEI, Polyplus-transfection Inc., New York, NY), control blank vector, p47^*phox*^ mutants S303A/S304A (dominant negative, DN) plasmid, AnxA2 plasmids (WT, C9A, C133A, C223A, C262A, and C335A), IL-17 plasmids (CRISPR/Cas9 KO sc-421092, CRISPR Activation sc-421092-ACT) (50 μg/mouse) 24 h before CLP procedures following the manufacturer's instruction [61,62].

### Oxidation assays

PMs isolated from lavage fluid were cultured in 96-well plates overnight. Neutrophils were isolated from the blood using radiopaque medium of differential density (Histopaque 1077 and 1119, Sigma). 3-(4,5-dimethylthiazol-2-yl)-2,5-dimethyltetrazolium bromide (MTT) assay, dihydro-dichlorofluorescein diacetate (H_2_DCF-DA, to detect reactive oxygen species assay, nitrobluetetrazolium (NBT) assay, and mitochondrial membrane potential (JC-1) assay were applied following the manufacturer’s instructions, respectively [63]. The levels of alanine aminotransferase (ALT) were determined by ALT activity assay kit (Cat#: MAK052, Sigma Aldrich) to evaluate hepatic injury and hepatic parenchymal damage. Lungs and other tissues were homogenized and equal protein amounts were used for myeloperoxidase (MPO), ALT or CFU assays.

### Histological analysis

After CLP, mouse colon, lung, kidney liver, and spleen tissues were fixed in 10% buffered formalin for 24 hours; and then processed for H&E staining operated by AML laboratories Inc. (Baltimore, MD); or immunostained with relevant primary antibodies.

### Flow cytometry

PMs were obtained from peritoneal fluid, and then single-cell suspensions were detected in Sham and CLP treated mice by flow cytometry as previously described [64]. Cells were washed by PBS three times and then incubated with indicated primary antibody for 30 min at 4°C. Data were analyzed and expressed as scattergrams using FlowJo 7.6 software [65].

### Enzyme-linked immunosorbent assay (ELISA)

Cytokine concentrations of KC, MCP-1, IFN-γ, TNF-α, IL-1β, IL-6, IL-17A, and IL-22 in peritoneal lavage fluid were measured by ELISA according to the manufacturer’s instructions (eBioscience, San Diego, CA).

### Gene expression profiling

Total RNA was extracted from peritoneal macrophages (PMs) with TRIzol reagents following the manufacturer’s instructions. After reverse transcription, real-time PCR profiling of mRNAs was conducted on a SYBR Green-based, RT² Profiler PCR Primer Assay Array System (Acute Inflammation Response, M384 Instrument: Bio-Rad CFX384, Cat#100–29814, Bio-Rad, Hercules, CA). Briefly, after initial incubation of 5 min at 95°C, 40 cycles include template denaturation (15s, 85°C) and followed annealing and elongation (30s, 65°C) under the C1000 Touch real-time PCR system [66]. Finally, data were analyzed by PrimePCR Analysis Application 1.0 software (Bio-Rad). NOX genes were also assayed and expressed as the fold difference to GAPDH using 2-ΔΔCT method, respectively (S2 Table).

### Immunoblotting

Samples taken from PMs or tissues after experimental treatment were lysed with radiation immunoprecipitation assay (RIPA) buffer (30 mM Tris-HCl, 150 mM NaCl, 2 mM EDTA, 1% Triton X-100, 10% glycerol), and complete protease inhibitor cocktail (Life technologies, Grand Island, NY) and phosphatase inhibitors (Sigma, St. Louis, MO). Lysates were centrifuged at 14000×g for 15 min, the supernatants were collected and the concentration was quantitated. The samples were boiled for 10 min, and equal amount was applied to 12% SDS-polyacrylamide minigels and electrophoresed. The proteins in the gel were then transferred to nitrocellulose filter membranes (Thermofisher, Rockford, IL). Horseradish peroxidase (HRP)-linked secondary antibody (Rockland, Gilbertsville, PA) was reacted with the membrane and X-ray film (Kodak) was used for exposure [67]. Mouse monoclonal IgG antibody anti-β-actin, p65, p-ERK and ERK, goat polyclonal IgG antibody anti-IL-1β, IL-6, and rabbit polyclonal IgG antibody anti-pp65, p38, and pp38 were bought from Santa Cruz Biotechnology (Santa Cruz, CA). Rabbit monoclonal IgG anti-Ly6G, NK1.1, and CD3 antibodies, mouse monoclonal IgG anti-F4/80, RORγt, IL-17A, and B220 antibodies were bought form Abcam (Cambridge, MA) [58].

### Statistics

Most experiments were conducted in triplicate. Differences between 2 groups were compared by one-way ANOVA (Tukey’s post hoc) using GraphPad Prism 5 software, while mice survival rates were calculated using Kaplan-Meier curves [65,68].

## Supporting Information

S1 Fig Analysis of cell populations in WT and *anxa2* ^*-/-*^ mice following CLP procedures. (A) Temporal changes in total number of cells recruited in the peritoneal cavity of WT mice and *anxa2* ^*-/-*^ mice. (B) Cumulative data for CD3^+^ (T cells) and B220^+^ (B cells) in the peritoneal cavity of WT and *anxa2* ^*-/-*^ mice. Means±SD from triplicate. (C) ELISA detecting cytokine secretion in peritoneal lavage from mice at 36, 48 and 72 h post-CLP, means±SD from triplicate. Data are representative of three independent experiments. One-way ANOVA (Tukey’s post hoc). *, p<0.05.
(TIF)

S2 Fig Serum and tissue ALT levels after CLP. (A) Ly6G^+^ and F4/80^+^ fluorescence scores in Fig 3A were quantified. (B) Mice were procedure for CLP for 24 h. PMs were isolated from peritoneal lavage for H_2_DCF and MPO assays after 1 h culturing. (C) ALT activity in different organs or tissues were assayed using ALT assay. Means+SD from triplicate. Data are representative from three independent experiments. One-way ANOVA (Tukey’s post hoc). *, p<0.05.
(TIF)

S3 Fig p47^*phox*^ S303A/S304A mutation dampens ROS production upon CLP treatment. (A) Peritoneal bacterial burdens were detected from *anxa2* ^*-/-*^ mice pre-injected with rotenone, DPI, APO, and NAC, respectively. (B) Mice were transfected with empty vector control or p47^*phox*^ S303A/S304A plasmid, then performed with CLP procedure for 24 h. Neutrophils from blood and PMs from peritoneal lavage were cultured for 1 h. ROS levels were determined using H_2_DCF assay. (C) Cell viabilities were measured using MTT assay. Means+SD from triplicate. Data are representative from three independent experiments. One-way ANOVA (Tukey’s post hoc). *, p<0.05; **, p<0.01.
(TIF)

S4 Fig DPI or H_2_O_2_ affects bacterial clearance during polymicrobial sepsis. (A) WT and *anxa2* ^*-/-*^ mice were pretreated with DPI or H_2_O_2_ and then subjected to CLP. At 24 h post-CLP, peritoneal lavage, blood and lung tissue were collected and performed for CFU assay to determine the bacterial burdens in mice treated as above. Data are shown as means±SD from 3 mice. One-way ANOVA (Tukey’s post hoc). (B) Kaplan-Meier survival curves from 6 mice in each group (Log-rank Test). *, p<0.05.
(TIF)

S5 Fig ROS affects IL-17 signaling during polymicrobial sepsis. (A, B) WT and *anxa2* ^*-/-*^ mice were pretreated with DPI or H_2_O_2_ and then subjected to CLP. At 24 h post-CLP, colon tissues were performed for paraffin histological analysis. Ly6G and IL-17A were used to detect neutrophils accumulation. Fluorescence scores were quantified as above. Data are representative from three independent experiments. Scale bar = 5 μm. (C) Mice were transfected with control blank or p47^*phox*^ S303A/S304A plasmid, then performed with CLP procedure for 24 h. IL-17A secretion in serum and peritoneal lavage was assayed by ELISA. (D) Bacterial burdens in peritoneal lavages were counted using CFU. Means+SD from triplicated. Data are representative from 3 independent experiments. One-way ANOVA (Tukey’s post hoc). *, p<0.05; **, p<0.01.
(TIF)

S6 Fig AnxA2 is associated with inflammatory cytokines release in bacterial sepsis models. (A) AnxA2 plasmids were tail vein injected to mice 24 h prior to CLP procedures, and AnxA2 protein abundance in colon tissue from both WT and *anxa2* ^*-/-*^ mice was measured using immunoblotting. Data are representative from 3 independent experiments. (B) *anxa2* ^*-/-*^ mice were then processed with CLP treatment. 24 h later, IL-17A, TNF-α, IL-6 and IL-22 were measured in peritoneal lavage. Means+SD from 3 mice. One-way ANOVA (Tukey’s post hoc). *, p<0.05; **, p<0.01.
(TIF)

S7 Fig AnxA2 is involved in ROS production in bacterial sepsis models. (A) ROS levels were determined in PMs from mice subjected to *E*. *coli* or *P*. *aeruginosa* (Pa)-induced sepsis (1×10^7^ CFU, intraperitoneal injection). (B) IL-17A secretion in peritoneal lavage was assayed by ELISA. (C, D) AnxA2 WT or indicated mutation plasmids were tail-vein injected to *anxa2* ^*-/-*^ mice 24 h before subjected to CLP, respectively. 24 h post-CLP, colon tissues were collected for immunostaining to detect IL-17A secretion. Florescence scores were quantified as above. Data are representative of three independent experiments. Scale bar = 5 μm. (E) AnxA2 WT or C9A plasmids were tail-vein injected to *anxa2* ^*-/-*^ mice 24 h before subjected to *E*. *coli* or Pa-induced sepsis, respectively. ROS levels were determined in PMs from mice using H_2_DCF assay. (F) ELISA assay was used to detect IL-17A accumulation in peritoneal lavage. Means±SD from triplicate. One-way ANOVA (Tukey’s post hoc); *, p<0.05; **, p<0.01.
(TIF)

S8 Fig IL-17 signaling is involved in polymicrobial sepsis. (A) Relative density of immunoblotting in Fig 7A was quantified and shown. (B) WT mice were pre-tail vein injected with IL-17 activation and KO plasmids, and 24 h later subjected to CLP-induced sepsis for 24 h. PMs isolated from peritoneal lavage were homogenized and subjected to qRT-PCR to detect IL-17A mRNA abundance. Data are representative from 3 independent experiments. (C) IL-17A mRNA abundance were measured in different tissues from above mice. (D) WT mice were tail vein injected with the IL-17 activation or KO plasmid, respectively. Ly6G^+^/F4/80^+^ events in peritoneal lavage were determined by flow cytometry. (E) WT mice and *anxa2* ^*-/-*^ mice were transfected with IL-17 plasmids as above, respectively. The mice were then subjected to CLP-induced sepsis for 24 h. Ly6G^+^/F4/80^+^ events in peritoneal lavage were quantified by flow cytometry. (F) Bacterial burdens in peritoneal lavage and blood were assayed by CFU. Data are representative and shown as means±SD from 3 mice. One-way ANOVA (Tukey’s post hoc); *, p<0.05.
(TIF)

S1 Table mRNA expression microarray analysis of peritoneal macrophages from mice. mRNA with greater than four-fold change were considered to be significantly regulated (NA, not available).
(DOCX)

S2 Table Primers used in amplification of targeted DNA. (DOCX)
