# Supplementary figures and images for: Annexin A2 Modulates ROS and Impacts Inflammatory Response via IL-17 Signaling in Polymicrobial Sepsis Mice
Source: PLoS Pathog. 2016 Jul 7;12(7):e1005743. doi: 10.1371/journal.ppat.1005743 (PMC4936746; doi:10.1371/journal.ppat.1005743)

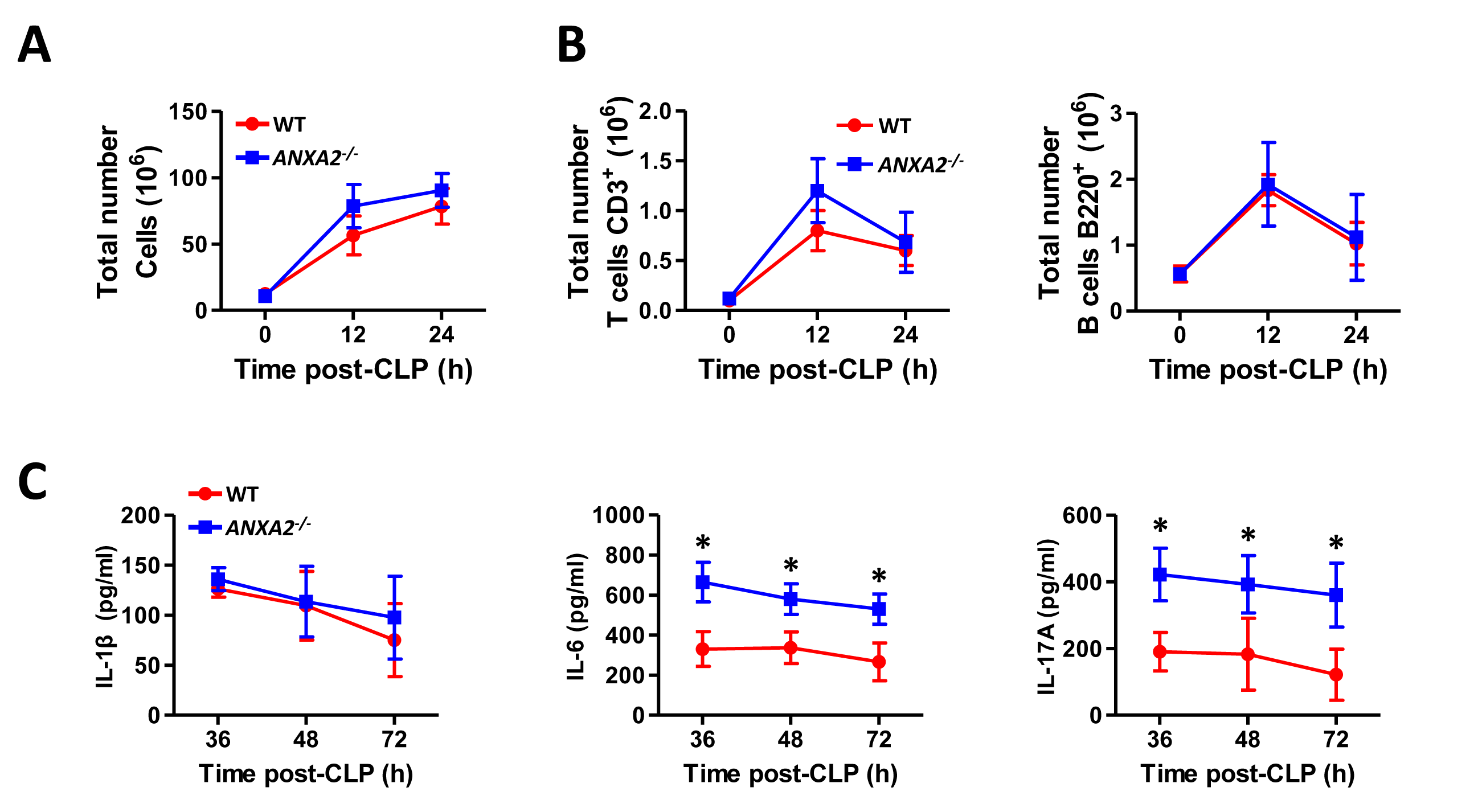

Supplement: S1 Fig — (A) Temporal changes in total number of cells recruited in the peritoneal cavity of WT mice and anxa2 -/- mice. (B) Cumulative data for CD3+ (T cells) and B220+ (B cells) in the peritoneal cavity of WT and anxa2 -/- mice. Means±SD from triplicate. (C) ELISA detecting cytokine secretion in peritoneal lavage from mice at 36, 48 and 72 h post-CLP, means±SD from triplicate. Data are representative of three independent experiments. One-way ANOVA (Tukey’s post hoc). *, p<0.05. (TIF) [file ppat.1005743.s001.tif]

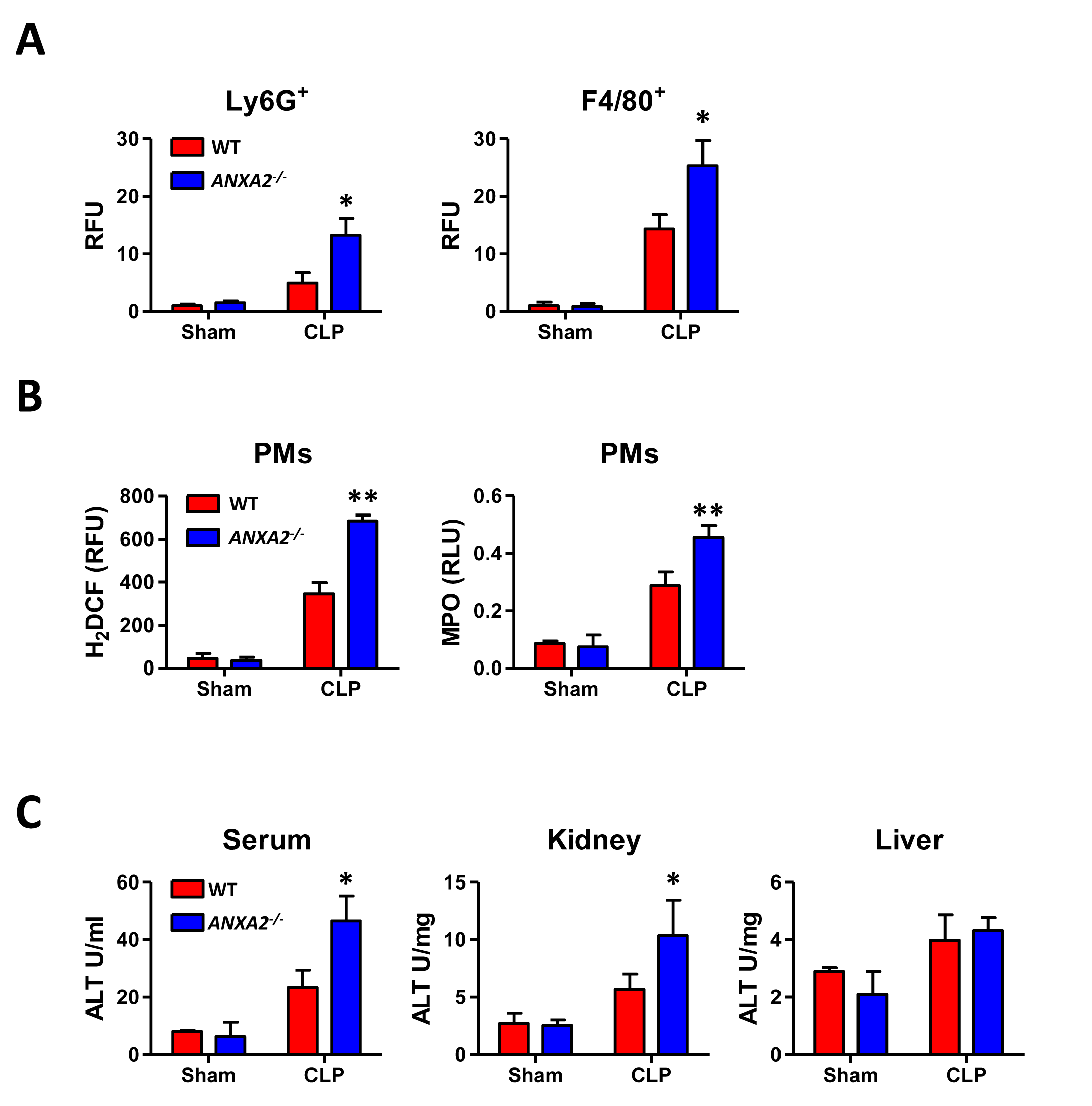

Supplement: S2 Fig — (A) Ly6G+ and F4/80+ fluorescence scores in Fig 3A were quantified. (B) Mice were procedure for CLP for 24 h. PMs were isolated from peritoneal lavage for H2DCF and MPO assays after 1 h culturing. (C) ALT activity in different organs or tissues were assayed using ALT assay. Means+SD from triplicate. Data are representative from three independent experiments. One-way ANOVA (Tukey’s post hoc). *, p<0.05. (TIF) [file ppat.1005743.s002.tif]

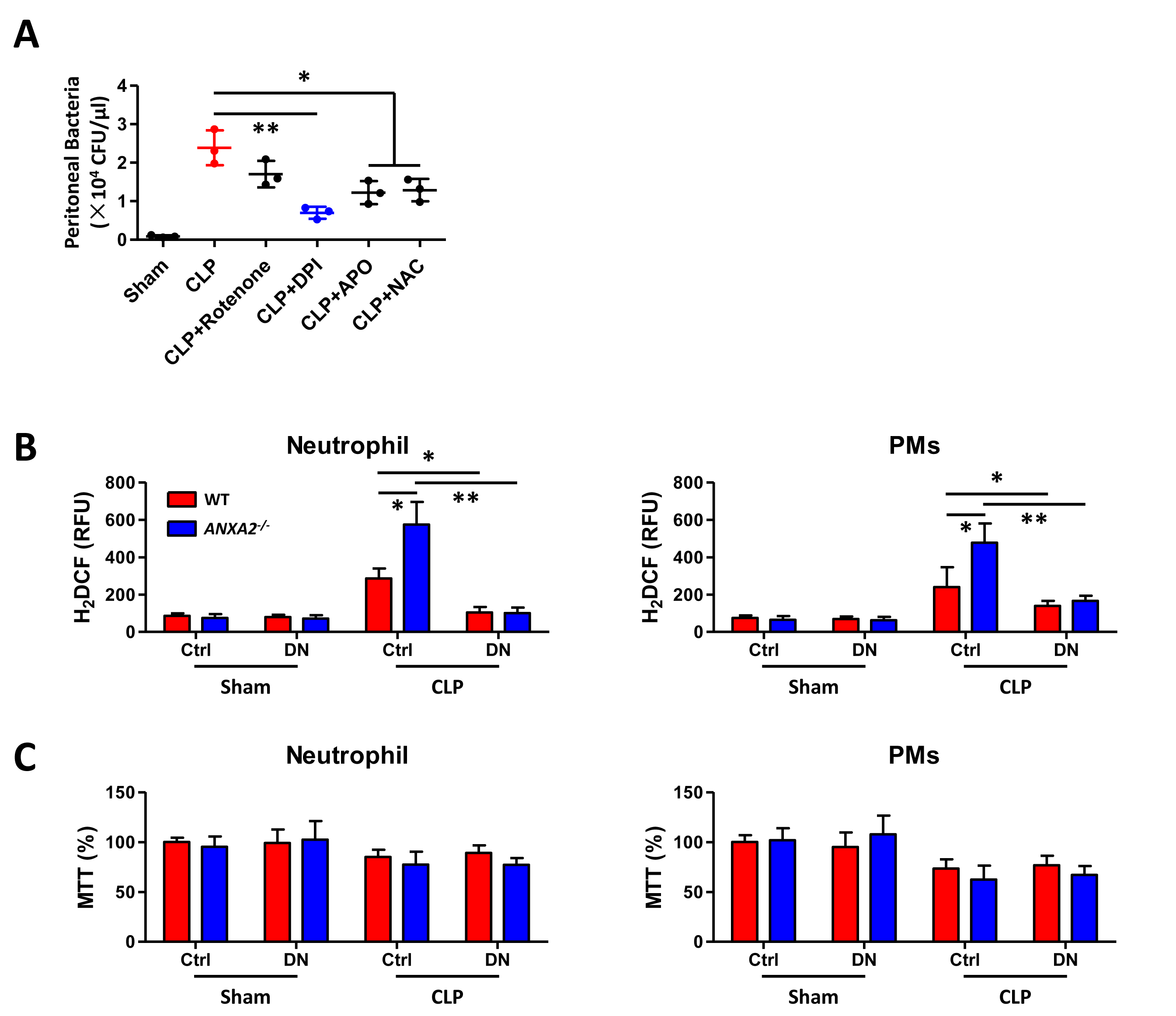

Supplement: S3 Fig — (A) Peritoneal bacterial burdens were detected from anxa2 -/- mice pre-injected with rotenone, DPI, APO, and NAC, respectively. (B) Mice were transfected with empty vector control or p47phox S303A/S304A plasmid, then performed with CLP procedure for 24 h. Neutrophils from blood and PMs from peritoneal lavage were cultured for 1 h. ROS levels were determined using H2DCF assay. (C) Cell viabilities were measured using MTT assay. Means+SD from triplicate. Data are representative from three independent experiments. One-way ANOVA (Tukey’s post hoc). *, p<0.05; **, p<0.01. (TIF) [file ppat.1005743.s003.tif]

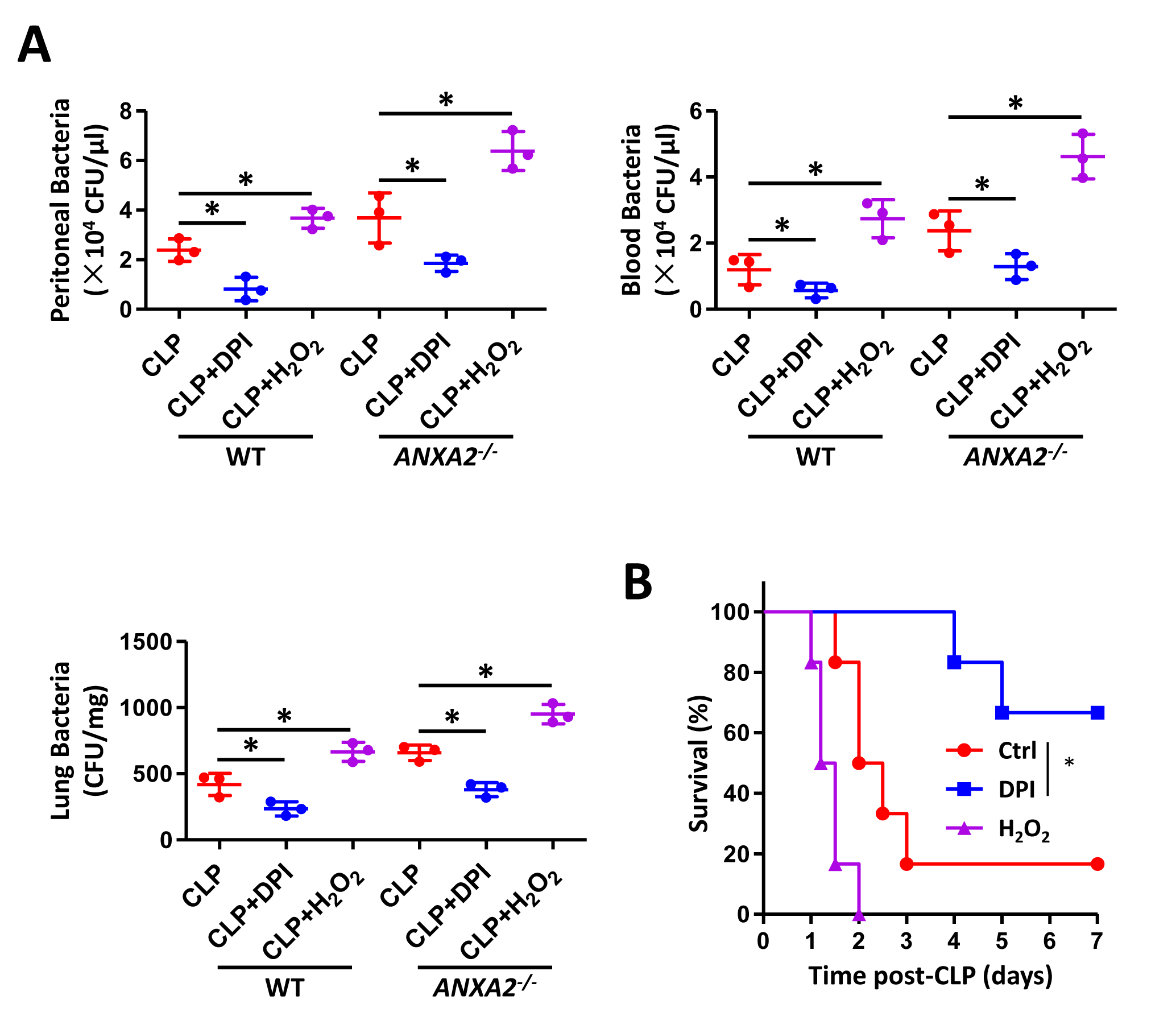

Supplement: S4 Fig — (A) WT and anxa2 -/- mice were pretreated with DPI or H2O2 and then subjected to CLP. At 24 h post-CLP, peritoneal lavage, blood and lung tissue were collected and performed for CFU assay to determine the bacterial burdens in mice treated as above. Data are shown as means±SD from 3 mice. One-way ANOVA (Tukey’s post hoc). (B) Kaplan-Meier survival curves from 6 mice in each group (Log-rank Test). *, p<0.05. (TIF) [file ppat.1005743.s004.tif]

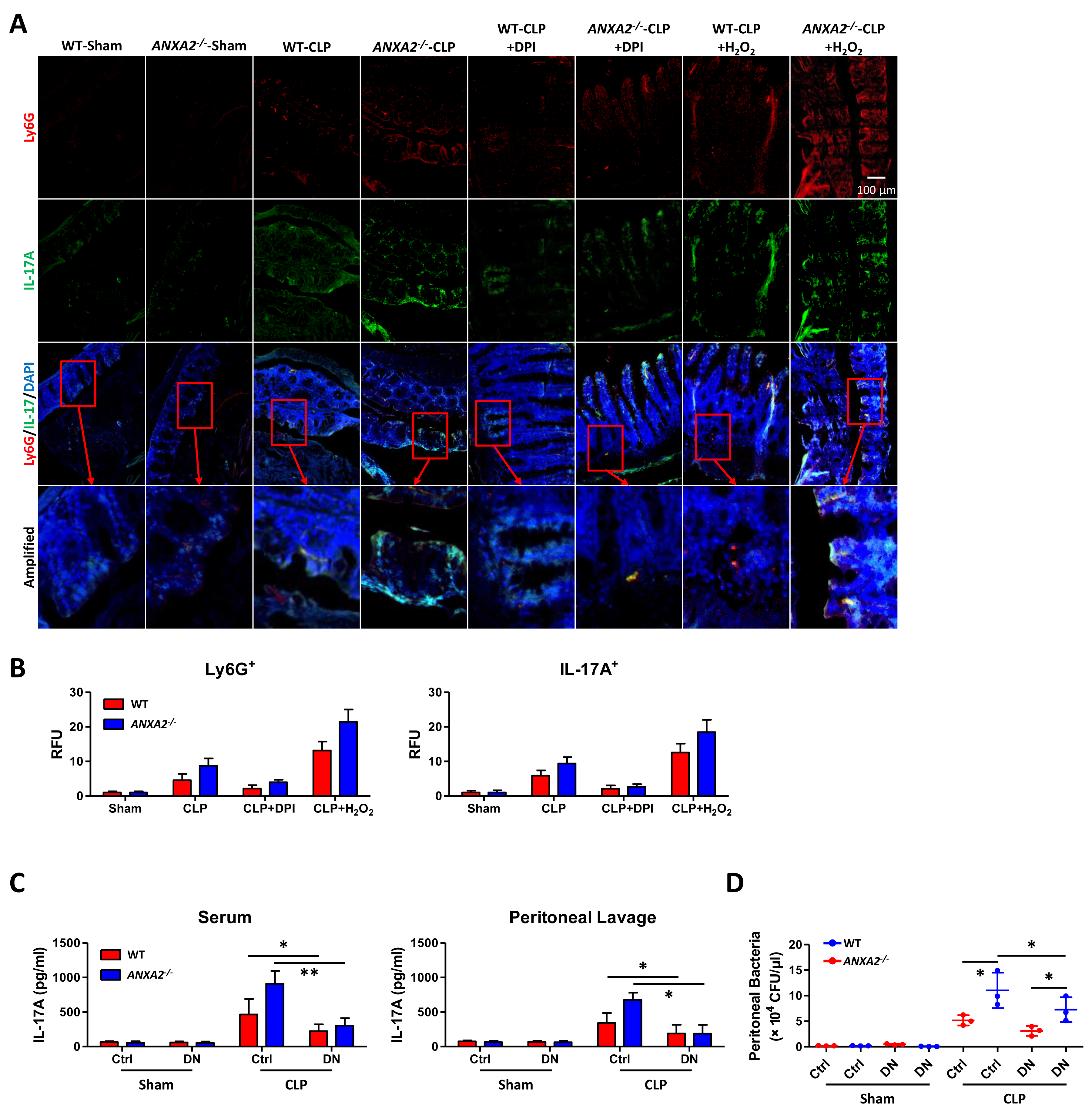

Supplement: S5 Fig — (A, B) WT and anxa2 -/- mice were pretreated with DPI or H2O2 and then subjected to CLP. At 24 h post-CLP, colon tissues were performed for paraffin histological analysis. Ly6G and IL-17A were used to detect neutrophils accumulation. Fluorescence scores were quantified as above. Data are representative from three independent experiments. Scale bar = 5 μm. (C) Mice were transfected with control blank or p47phox S303A/S304A plasmid, then performed with CLP procedure for 24 h. IL-17A secretion in serum and peritoneal lavage was assayed by ELISA. (D) Bacterial burdens in peritoneal lavages were counted using CFU. Means+SD from triplicated. Data are representative from 3 independent experiments. One-way ANOVA (Tukey’s post hoc). *, p<0.05; **, p<0.01. (TIF) [file ppat.1005743.s005.tif]

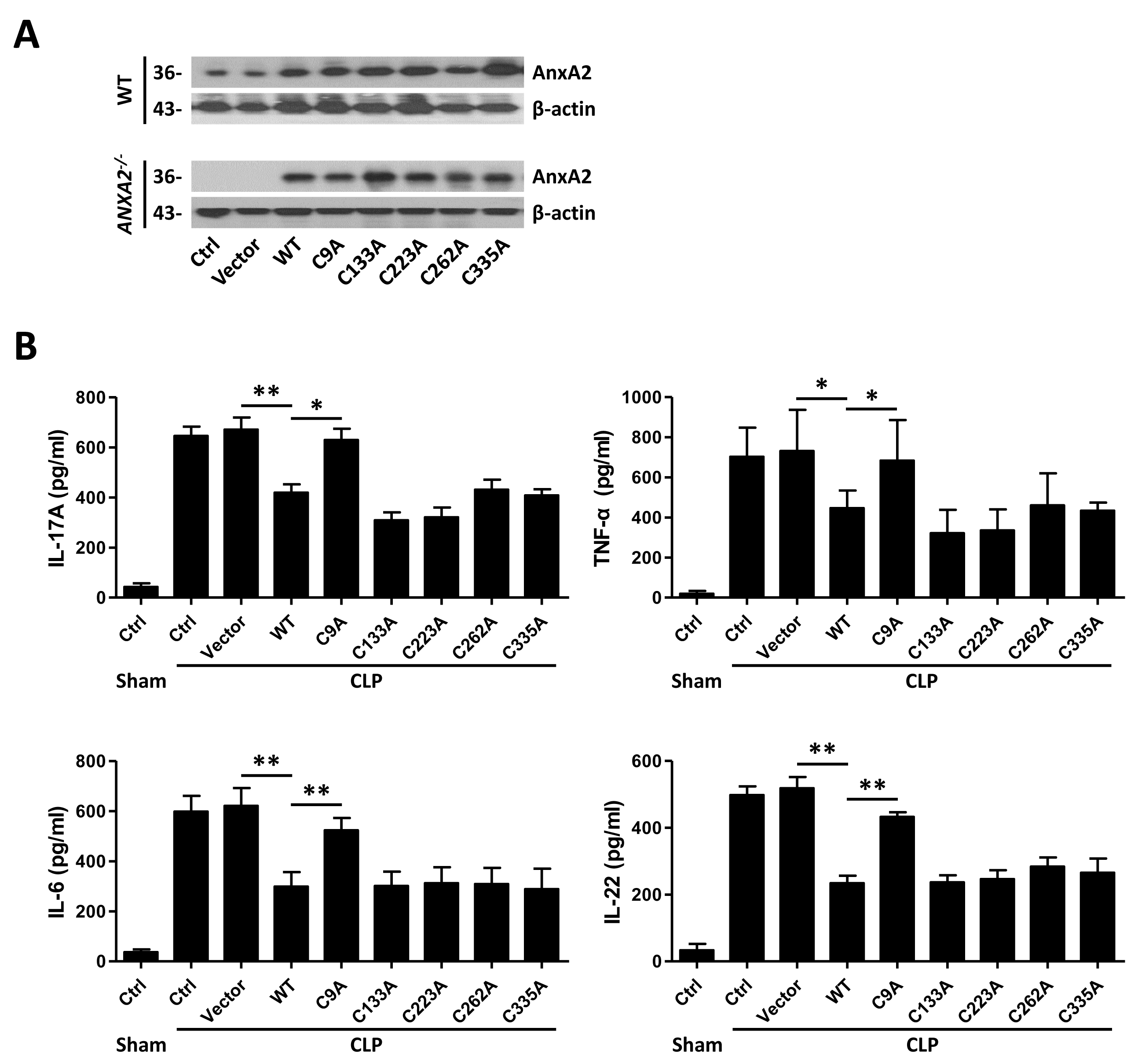

Supplement: S6 Fig — (A) AnxA2 plasmids were tail vein injected to mice 24 h prior to CLP procedures, and AnxA2 protein abundance in colon tissue from both WT and anxa2 -/- mice was measured using immunoblotting. Data are representative from 3 independent experiments. (B) anxa2 -/- mice were then processed with CLP treatment. 24 h later, IL-17A, TNF-α, IL-6 and IL-22 were measured in peritoneal lavage. Means+SD from 3 mice. One-way ANOVA (Tukey’s post hoc). *, p<0.05; **, p<0.01. (TIF) [file ppat.1005743.s006.tif]

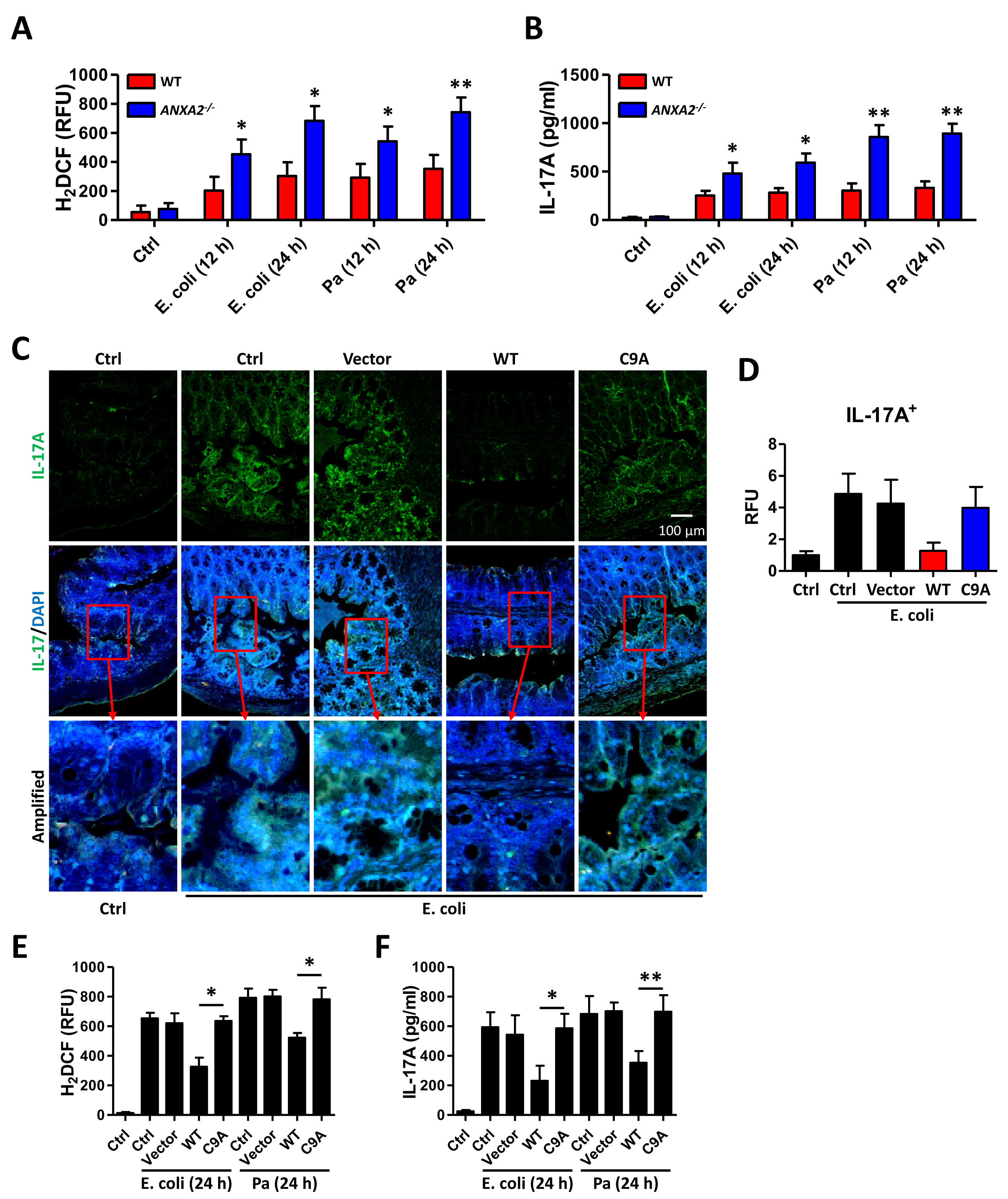

Supplement: S7 Fig — (A) ROS levels were determined in PMs from mice subjected to E. coli or P. aeruginosa (Pa)-induced sepsis (1×10^7^ CFU, intraperitoneal injection). (B) IL-17A secretion in peritoneal lavage was assayed by ELISA. (C, D) AnxA2 WT or indicated mutation plasmids were tail-vein injected to anxa2 -/- mice 24 h before subjected to CLP, respectively. 24 h post-CLP, colon tissues were collected for immunostaining to detect IL-17A secretion. Florescence scores were quantified as above. Data are representative of three independent experiments. Scale bar = 5 μm. (E) AnxA2 WT or C9A plasmids were tail-vein injected to anxa2 -/- mice 24 h before subjected to E. coli or Pa-induced sepsis, respectively. ROS levels were determined in PMs from mice using H2DCF assay. (F) ELISA assay was used to detect IL-17A accumulation in peritoneal lavage. Means±SD from triplicate. One-way ANOVA (Tukey’s post hoc); *, p<0.05; **, p<0.01. (TIF) [file ppat.1005743.s007.tif]

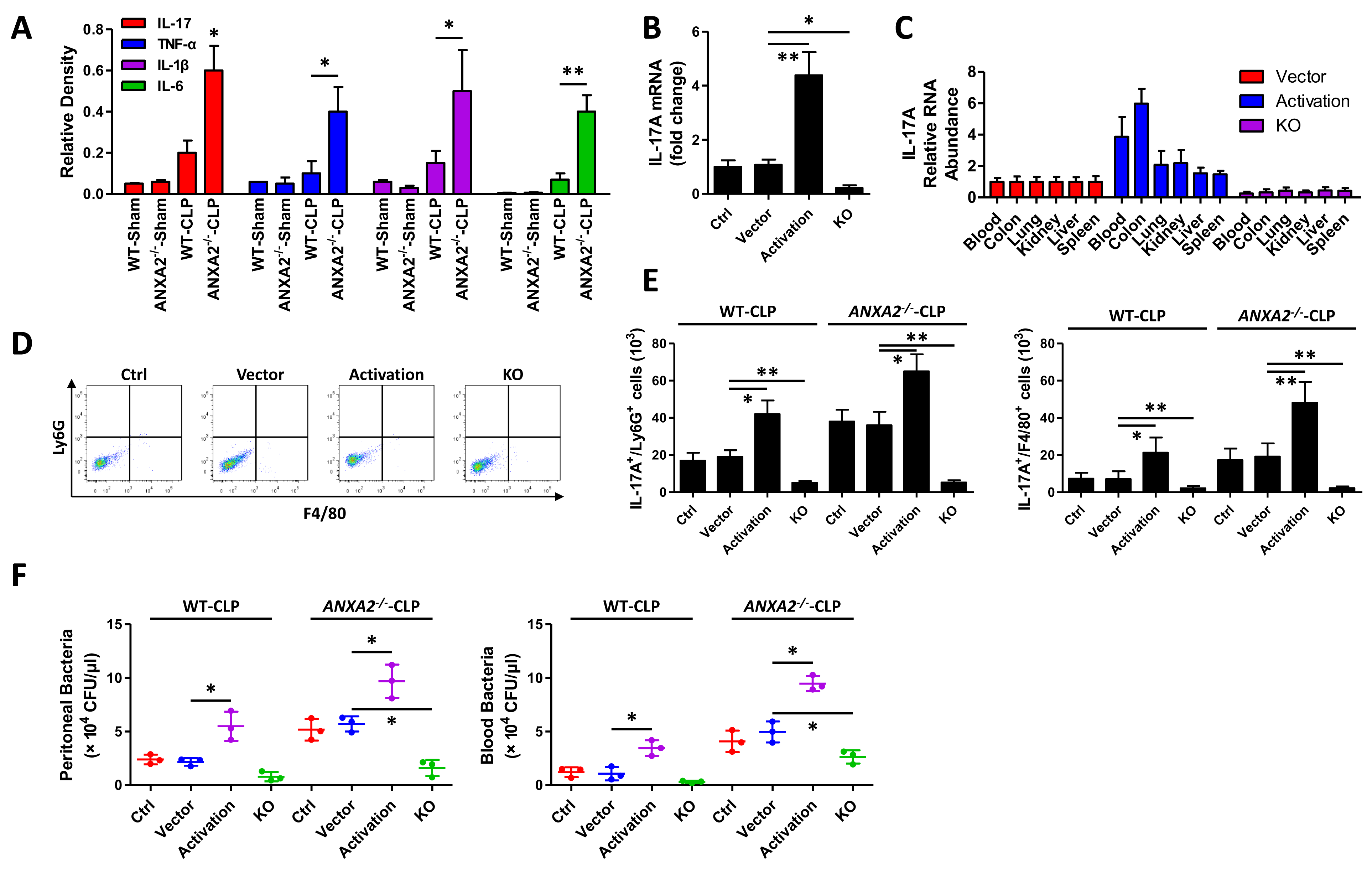

Supplement: S8 Fig — (A) Relative density of immunoblotting in Fig 7A was quantified and shown. (B) WT mice were pre-tail vein injected with IL-17 activation and KO plasmids, and 24 h later subjected to CLP-induced sepsis for 24 h. PMs isolated from peritoneal lavage were homogenized and subjected to qRT-PCR to detect IL-17A mRNA abundance. Data are representative from 3 independent experiments. (C) IL-17A mRNA abundance were measured in different tissues from above mice. (D) WT mice were tail vein injected with the IL-17 activation or KO plasmid, respectively. Ly6G+/F4/80+ events in peritoneal lavage were determined by flow cytometry. (E) WT mice and anxa2 -/- mice were transfected with IL-17 plasmids as above, respectively. The mice were then subjected to CLP-induced sepsis for 24 h. Ly6G+/F4/80+ events in peritoneal lavage were quantified by flow cytometry. (F) Bacterial burdens in peritoneal lavage and blood were assayed by CFU. Data are representative and shown as means±SD from 3 mice. One-way ANOVA (Tukey’s post hoc); *, p<0.05. (TIF) [file ppat.1005743.s008.tif]
