# Supplementary material for: Annexin A2 Modulates ROS and Impacts Inflammatory Response via IL-17 Signaling in Polymicrobial Sepsis Mice
Source: PLoS Pathog. 2016 Jul 7;12(7):e1005743. doi: 10.1371/journal.ppat.1005743 (PMC4936746; doi:10.1371/journal.ppat.1005743)
Supplement: S1 Table — mRNA with greater than four-fold change were considered to be significantly regulated (NA, not available). (DOCX) [file ppat.1005743.s009.docx]

**Supplementary Table 1.** mRNA expression microarray analysis of peritoneal macrophages from mice. mRNA with greater than four-fold change were considered to be significantly regulated (NA, not available).

| **Gene Symbol** | **ANXA2^-/-^-Sham VS WT-Sham Fold Change** | **WT-CLP VS WT-Sham Fold Change** | **ANXA2^-/-^-CLP VS WT-CLP Fold Change** |
| --- | --- | --- | --- |
| A2m | NA | NA | NA |
| Apoa1 | 1.22307 | 3.54301 | 2.22099 |
| Apoe | 1.56550 | -1.30899 | 1.40904 |
| Bdkrb1 | NA | NA | -1.11917 |
| C3ar1 | 1.53531 | 1.37532 | 2.13373 |
| C5ar1 | NA | NA | NA |
| Calca | 2.32709 | -2.14389 | 3.37146 |
| Ccl12 | -1.11809 | 1.21456 | 7.14380 |
| Ccr1 | -1.83118 | 3.19232 | -1.11917 |
| Ccr2 | 3.62904 | 1.86964 | 2.59384 |
| Ccr3 | NA | NA | NA |
| Ccr5 | 3.00546 | 4.47986 | 7.20963 |
| Ccr6 | 2.32989 | 1.27302 | 1.78337 |
| Ccr7 | 1.35522 | -1.11747 | 4.63759 |
| Ccr8 | NA | NA | NA |
| Cd14 | -19.82713 | NA | NA |
| Cd163 | -2.60411 | 1.66489 | 1.69950 |
| Cd4 | NA | NA | NA |
| Cebpb | -8.22980 | 1.73329 | -1.30662 |
| Crp | 2.74828 | -5.80457 | 15.95385 |
| Cx3cr1 | 4.14474 | 2.95733 | 1.97829 |
| Cxcl1 | -7.55449 | 7.60355 | NA |
| Cxcl10 | 1.36309 | 9.09879 | 9.19020 |
| Cxcr1 | NA | NA | NA |
| Cxcr2 | NA | NA | NA |
| Cxcr3 | 2.70963 | 1.09396 | 3.79134 |
| Cxcr4 | 4.47967 | -1.86948 | 4.93792 |
| Cxcr6 | 2.86326 | -1.21756 | 1.99104 |
| Defb1 | NA | NA | NA |
| Elane | 1.74723 | 3.44751 | 1.93737 |
| F2 | NA | NA | NA |
| F2r | NA | NA | NA |
| F2rl1 | 2.29208 | 1.38717 | 2.76386 |
| F3 | 1.43925 | 1.07407 | 2.42377 |
| Fcgr1 | 1.83965 | 2.50396 | 1.75225 |
| Fpr2 | -9.01567 | -2.31229 | 1.72951 |
| Gata3 | 1.61671 | -2.39534 | 6.75472 |
| Gm20425 | NA | NA | NA |
| Hba-a1 | 1.63898 | 1.13132 | 2.49471 |
| Hba-a2 | 1.51186 | 1.13315 | 1.77884 |
| Hmgb1 | 2.25438 | 1.05029 | 1.34890 |
| Hp | 2.67153 | -1.41791 | 2.67991 |
| Icam1 | 2.25109 | 2.31652 | 2.08919 |
| Ifng | NA | NA | NA |
| Il10 | 2.62182 | 2.21057 | 6.48094 |
| Il13 | 1.91128 | 3.71654 | 2.39430 |
| **Il17a** | 1.50341 | **8.17526** | **16.07355** |
| Il18 | 2.41376 | 4.33799 | 1.26847 |
| Il1a | 2.80215 | 1.23829 | 8.40795 |
| Il1b | -2.22297 | 6.60138 | 2.39774 |
| Il1rn | NA | NA | NA |
| Il2 | 2.34870 | 5.41854 | 2.49445 |
| Il22 | NA | NA | NA |
| Il4 | 1.19522 | 5.66489 | 5.12463 |
| Il5 | NA | NA | NA |
| Il6 | NA | NA | NA |
| Il6ra | NA | NA | NA |
| Il6st | -2.26808 | -2.09977 | 2.77671 |
| Iltifb | 1.37792 | -2.01117 | 2.16927 |
| Ins2 | NA | NA | NA |
| Itgam | 1.76871 | -1.74025 | 4.16936 |
| Lbp | 1.47974 | 1.26896 | 1.42231 |
| Mapk1 | 2.15249 | 1.07139 | 1.61456 |
| Mbl2 | NA | NA | NA |
| Mpo | 1.21005 | 2.64996 | 2.58509 |
| Nfkb1 | 1.42810 | 1.98021 | 2.10361 |
| Ptges | NA | NA | NA |
| Rela | -4.98821 | -1.79034 | -4.63759 |
| S100a8 | 2.07696 | -1.57843 | 3.80988 |
| S100a9 | 2.80686 | 2.10377 | 2.03307 |
| Saa1 | -13.86925 | 3.46191 | 1.41400 |
| Saa2 | -7.77434 | -2.16895 | 1.30628 |
| Serpina1a | NA | NA | NA |
| Serpina1b | 1.08499 | -1.56921 | -10.01763 |
| Serpina1c | -1.24672 | 1.99187 | -1.10361 |
| Serpina1d | NA | NA | NA |
| Serpina1e | 1.92153 | -1.09272 | 2.63054 |
| Serpina3n | NA | NA | NA |
| Sigirr | 3.36502 | 1.67491 | -1.26242 |
| Stat3 | 2.39976 | 3.01391 | 2.73842 |
| Tfrc | 2.90029 | -1.26221 | 2.92220 |
| Tgfb1 | 1.69187 | 12.37004 | 11.68154 |
| Tlr2 | 1.37535 | 5.88927 | 2.05896 |
| Tlr4 | 1.80069 | 11.64020 | 1.74344 |
| Tnf | 1.02771 | 1.22897 | 15.66781 |
| Tnfrsf1a | 2.98665 | 2.20346 | 1.40773 |
| Tnfsf4 | NA | NA | NA |
| Vcam1 | 1.89912 | 1.43100 | 3.54890 |
