# Supplementary material for: Annexin A2 Modulates ROS and Impacts Inflammatory Response via IL-17 Signaling in Polymicrobial Sepsis Mice
Source: PLoS Pathog. 2016 Jul 7;12(7):e1005743. doi: 10.1371/journal.ppat.1005743 (PMC4936746; doi:10.1371/journal.ppat.1005743)
Supplement: S2 Table — (DOCX) [file ppat.1005743.s010.docx]

**Supplementary Table 2.** Primers used in amplification of targeted DNA (highlighted indicate restriction sites or amino acid mutations).

| **ID** | **Primer Sequences (5’-3’)** |
| --- | --- |
| ANXA2 Forward | GTGCCTACGGGTCAGTCAAA |
| ANXA2 Reverse | CACATTGCTGCGGTTTGTCA |
| NOX1 Forward | CTGCTGTCCTTCTTGAGGGG |
| NOX1 Reverse | GCCACCAGCTTATGGAAGGT |
| NOX3 Forward | GTGTGCATTGCAACGGGAAT |
| NOX3 Reverse | GCATCCCGGCAGATCCAATA |
| NOX4 Forward | TGGCCAACGAAGGGGTTAAA |
| NOX4 Reverse | GATGAGGCTGCAGTTGAGGT |
| GAPDH Forward | CAGGTTGTCTCCTGCGACTT |
| GAPDH Reverse | TATGGGGGTCTGGGATGGAA |
| ANXA2 WT Forward | GAG**CTGCAG**ATGTCTACTGTCCACGAAATC |
| ANXA2 WT Reverse | GTA**TCTAGA**TCAGTCATCCCCACCACACAG |
| ANXA2 C9A Forward | GAT**CTGCAG**ATGTCTACTGTCCACGAAATCCTGGCCAAGCTCAGCCTGGA |
| ANXA2 C133A Forward | TCATTGAGATCATCGCCTCACGAACCAACC |
| ANXA2 C133A Reverse | GGTTGGTTCGTGAGGCGATGATCTCAATGA |
| ANXA2 C223A Forward | TGAGCGCAGTGTGGCCCACCTCCAGAAA |
| ANXA2 C223A Reverse | TTTCTGGAGGTGGGCCACACTGCGCTCA |
| ANXA2 C262A Forward | TGAACCTGGTCCAGGCCATCCAGAACAAGC |
| ANXA2 C262A Reverse | GCTTGTTCTGGATGGCCTGGACCAGGTTCA |
| ANXA2 C335A Reverse | CTA**TCTAGA**TCAGTCATCCCCACCAGCCAGGTACAGCAGTG |
